# Consistent supra­molecular motifs and different local symmetries in the structures of 2-amino-5-(4-fluoro­phen­yl)-1,3-thia­zole-4-carbaldehyde and 2-amino-5-(4-chloro­phen­yl)-1,3-thia­zole-4-carbaldehyde

**DOI:** 10.1107/S2056989025010667

**Published:** 2026-01-01

**Authors:** Firudin I. Guseinov, Ksenia A. Afanaseva, Sergey M. Gaidar, Anna M. Pikina, Mehmet Akkurt, Fargana S. Aliyeva, Khudayar I. Hasanov, Alebel N. Belay

**Affiliations:** a Kosygin State University of Russia, 117997 Moscow, Russian Federation; b N.D. Zelinsky Institute of Organic Chemistry, Russian Academy of Sciences, 119991 Moscow, Russian Federation; c Russian State Agrarian University-Moscow Timiryazev Agricultural Academy 127550 Moscow Russian Federation; d Department of Physics, Faculty of Sciences, Erciyes University, 38039 Kayseri, Türkiye; e Excellence Center, Baku State University, Z. Xalilov Str. 23, Az 1148 Baku, Azerbaijan; f Azerbaijan Medical University, Scientific Research Centre (SRC), Kasumzade St. 14. AZ 1022, Baku, Azerbaijan; g Department of Chemistry, Bahir Dar University, PO Box 79, Bahir Dar, Ethiopia; University of Aberdeen, United Kingdom

**Keywords:** crystal structure, 1,3-thia­zole ring, haloketones, halooxiranes

## Abstract

The first title compound, C_10_H_7_FN_2_OS, crystallizes in space group *P*1 with two independent mol­ecules in the asymmetric unit, which form a dimer with an *R*^2^_2_(8) motif through pairwise N—H⋯N hydrogen bonds. In the crystal of (I), N—H⋯O hydrogen bonds bind the dimers into zigzag ribbons running along the [100] direction, generating *R*^4^_4_(14) motifs. The second title compound, C_10_H_7_ClN_2_OS (space group *I*2/*a*), contains one mol­ecule in the asymmetric unit, which forms a dimer with an *R*^2^_2_(8) motif *via* inversion symmetry. In the extended structure, the mol­ecules form zigzag ribbons in the [100] direction by N—H⋯N and N—H⋯O hydrogen bonds, resulting in consecutive *R*^4^_1_(8)*R*^2^_1_(5)*R*^2^_2_(8)*R*^2^_1_(5)*R*^4^_1_(8) motifs. The Hirshfeld surface analyses of the compounds (I) and (II) indicates that the most important factors influencing the crystal packing are H⋯H inter­actions [21.1% for mol­ecule *A* of (I), 20.3% for mol­ecule *B* of (I) and 21.0% for (II)].

## Abstract 2

The title compounds have different local symmetries but a consistent supra­molecular motif of zigzag supra­molecular ribbons propagating along the [100] direction linked by N—H⋯N and N—H⋯O hydrogen bonds. Various weak inter­actions consolidate both structures.

## 1. Chemical context

Thia­zole and its derivatives are known for their broad spectrum of biological applications, such as anti­microbial, anti-inflammatory, anti­viral, anti­tubercular and CNS active agents and for their anti­cancer activities (Basarab *et al.*, 2012 ▸; Shaikh *et al.*, 2023 ▸). It should be noted that the thia­zole-4-carbaldehyde fragment is part of the natural polyketide thuggacin A, which has high anti-tuberculosis activity (Liu *et al.*, 2025 ▸). Earlier, we showed that acetal-containing chloro­oxiranes and their isomeric chloro­ketones are effective starting reagents for obtaining heterocyclic systems, in particular, heterocyclic aldehydes (Guseinov & Yudina, 1998 ▸; Guseinov *et al.*, 2017 ▸).
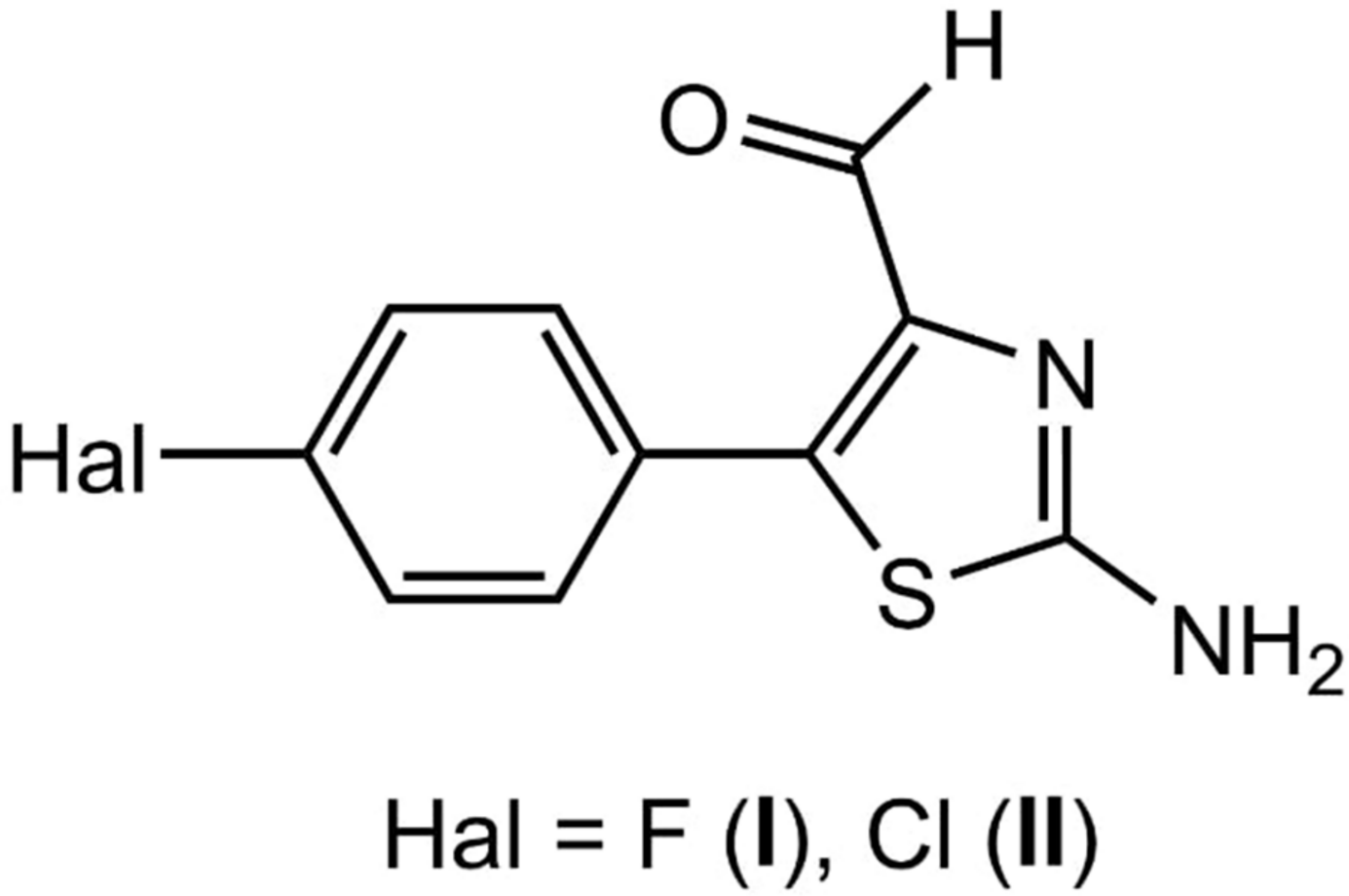


In this work, we describe a one-step synthetic protocol to access 2-amino-5-(4-halophen­yl)thia­zole-4-carbaldehydes and a study of their structural features using X-ray diffraction.

## 2. Structural commentary

**Figure 1:**
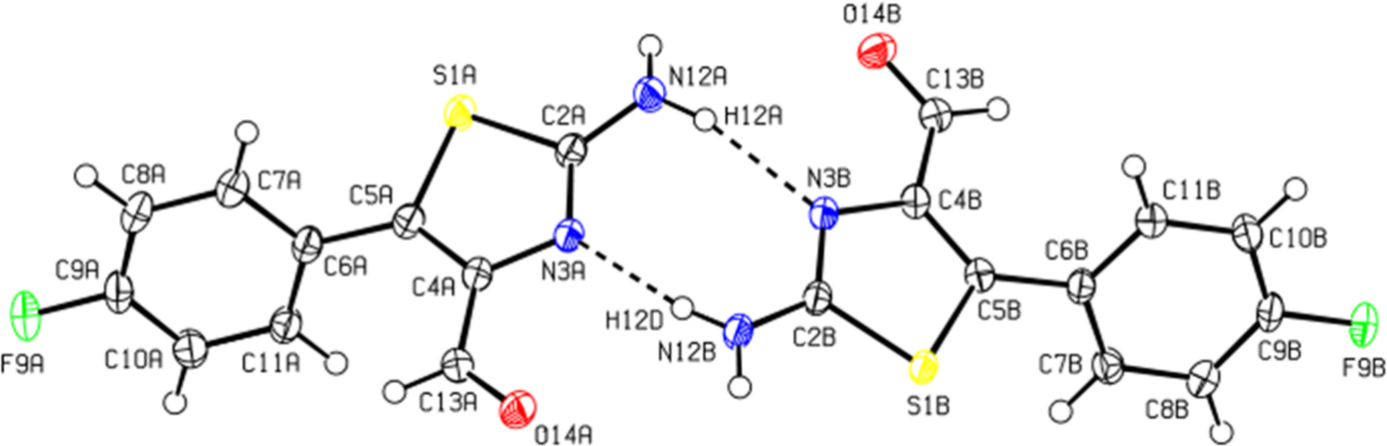
The asymmetric unit of (I) with displacement ellipsoids drawn at the 50% probability level.

Compound (I) crystallizes in the triclinic space group *P*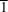 with two crystallographically independent mol­ecules, *A* and *B*, in the asymmetric unit (Fig. 1 ▸). An overlay fit of inverted mol­ecule *B* on mol­ecule *A* is shown in supplementary Fig. S1: the weighted r.m.s. fit of the 15 non-H atoms being 0.114 Å with the major differences being in the terminal benzene rings of mol­ecules *A* and *B*. The dihedral angle between the planes of the five and six-membered rings is 61.74 (8)° for *A* and 57.07 (8)° for *B*. Selected bond lengths include C9*A*—F9*A* = 1.3580 (18) Å for mol­ecule *A* and C9*B*—F9*B* = 1.3626 (17) Å for mol­ecule B. The C—N bond lengths in the five-membered rings are C2*A*—N3*A* = 1.308 (2) and C4*A*—N3*A* = 1.384 (2) Å for *A* and C2*B*—N3*B* = 1.309 (2) and C4*B*—N3*B* = 1.388 (2) Å for *B*. The C—N bond length attached to the five-membered ring of the NH_2_ group is C2*A*—N12*A* = 1.343 (2) Å for *A* and C2*B*—N12*B* = 1.344 (2) Å for *B*.

**Figure 2:**
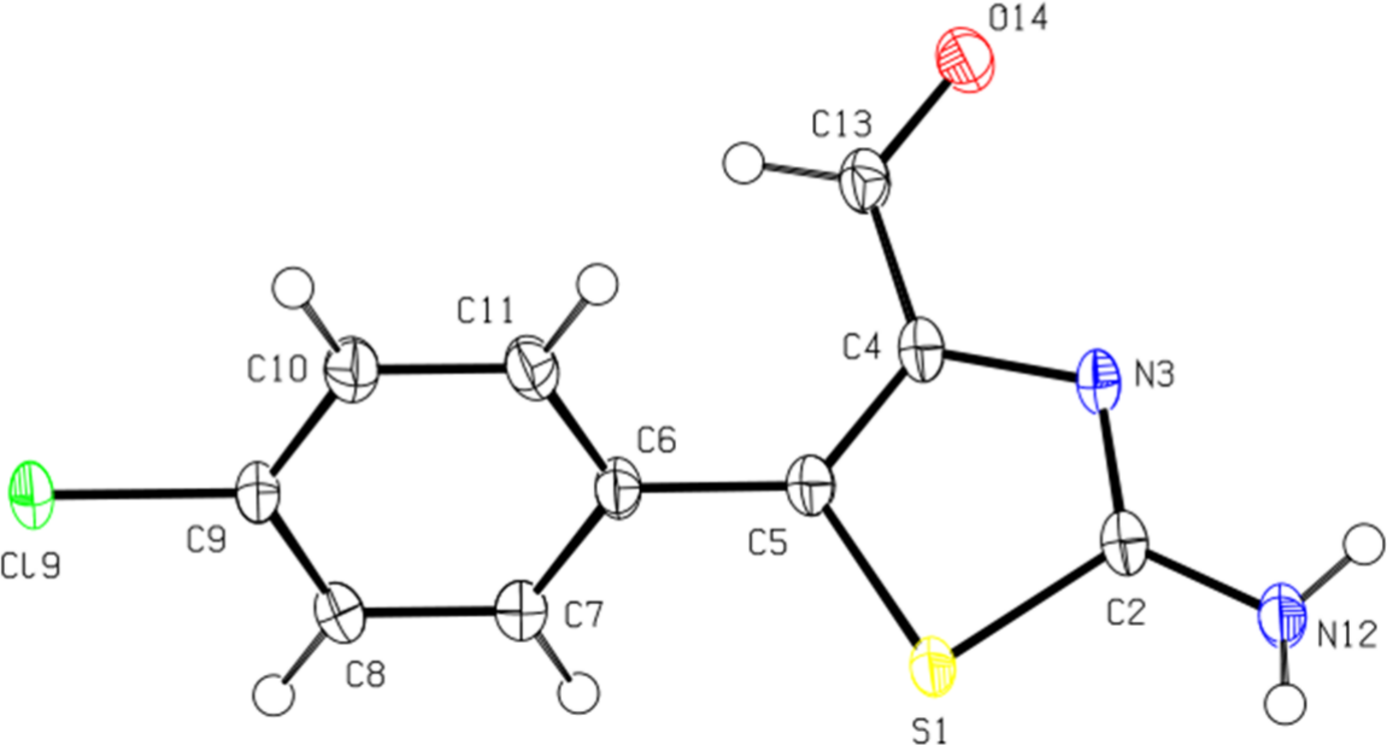
The asymmetric unit of (II) with displacement ellipsoids drawn at the 50% probability level.

Compound (II), which crystallizes in space group *I*2/*a* with one mol­ecule in the asymmetric unit (Fig. 2 ▸) has a non planar conformation in which the dihedral angle between the planes of the benzene and 1,3-thia­zole rings is 56.50 (8)°. The torsion angles S1—C5—C6—C7 and C4—C5—C6—C11 are −57.7 (2) and −53.7 (3)°, respectively. The C—N lengths [C2—N3 and C4—N3] in the five-membered ring are 1.307 (2) and 1.386 (2) Å, respectively. The C—N length [C2—N12] for the NH_2_ group attached to the ring is 1.346 (2) Å and the C9—Cl9 bond length is 1.7410 (16) Å.

Otherwise, the bond lengths and angles in compounds (I) and (II) are normal and can be compared with each other and with those in the *Database Survey* section.

## 3. Supra­molecular features and Hirshfeld surface analyses

**Figure 3:**
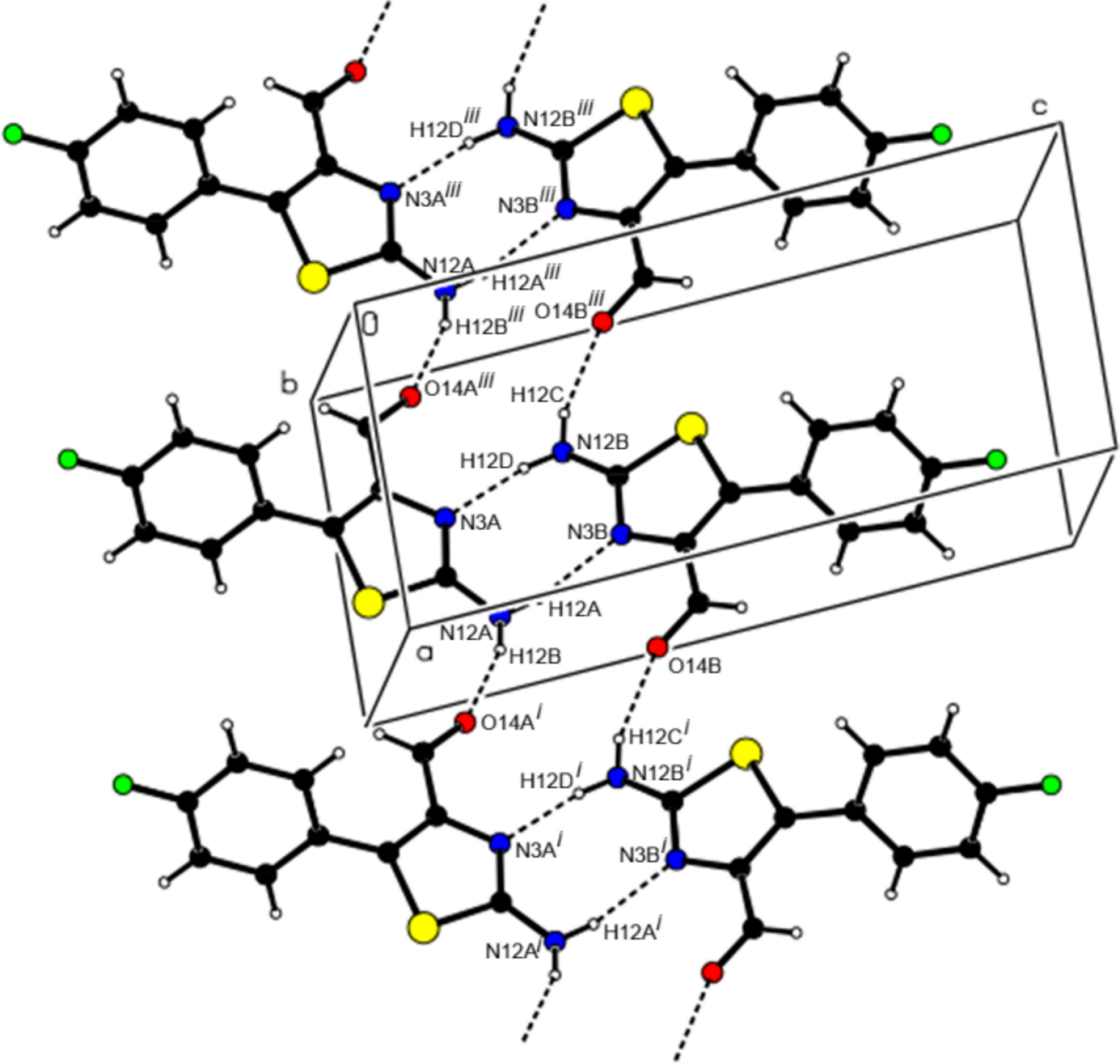
Partial packing diagram for (I) showing ribbons extending along the [100] direction with an [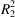
(8)
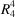
(14)]_*n*_ motif formed by N—H⋯N and N—H⋯O hydrogen bonds. Symmetry codes: (i) *x* + 1, *y*, *z*; (iii) *x -* 1, *y*, *z*.

The two independent mol­ecules (*A* and *B*) in the asymmetric unit of (I) form a dimer with an 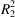
(8) motif through pairwise N—H⋯N hydrogen bonds (Table 1 ▸). In the crystal, N—H⋯O hydrogen bonds link the dimers into zigzag ribbons extending along the [100] direction, producing 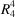
(14) motifs between them (Fig. 3 ▸). Additionally, the mol­ecules in these ribbons form 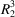
(11) motifs through C—H⋯S and C—H⋯F inter­actions, resulting in a three-dimensional supra­molecular network. A weak C—H⋯π inter­action also occurs (supplementary Figs. S2–S4).

**Figure 4:**
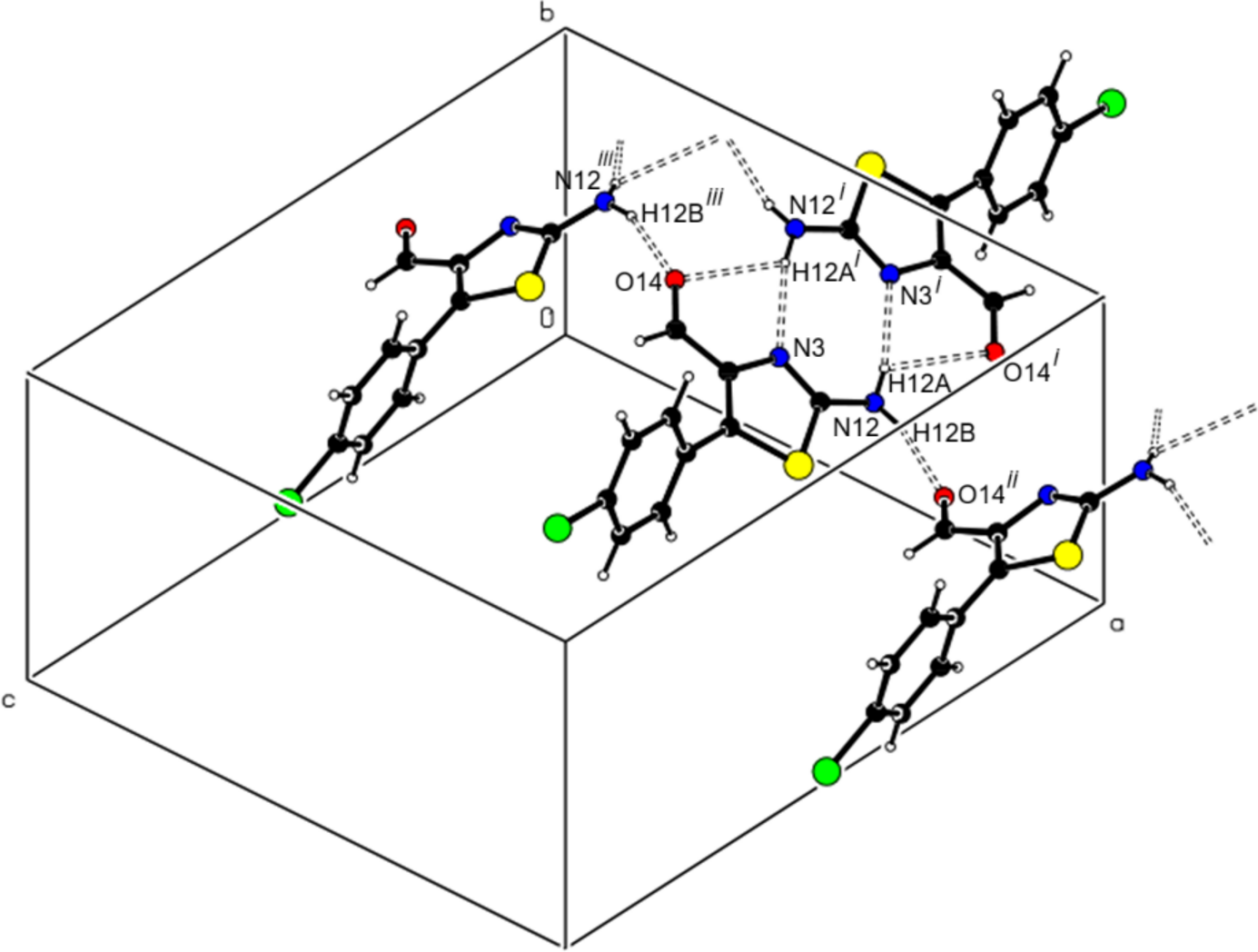
A partial view of the packing of (II) showing N—H⋯N and N—H⋯O hydrogen bonds, forming zigzag ribbons propagating along the [100] direction, with successive [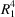
(8)
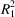
(5)
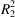
(8)
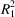
(5)
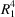
(8)]_*n*_ motifs. Symmetry codes: (i) −*x* + 1, −*y* + 1, −*z*; (ii) *x* + 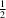, −*y* + 1, *z*; (iii) *x* + 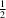, −*y* + 1, *z*.

In the extended structure of (II), the mol­ecules are linked through N—H⋯N and N—H⋯O hydrogen bonds (Table 2 ▸), forming zigzag ribbons propagating along the [100] direction, generating successive 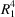
(8)
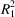
(5)
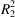
(8)
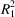
(5)
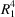
(8) motifs (Fig. 4 ▸). In addition, π–π [*Cg*2⋯*Cg*2^*a*^ = 3.8099 (11) Å, slippage = 1.011 Å; symmetry code (*a*) 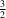 − *x*, *y*, 1 − *z*; *Cg*2 is the centroid of the (C6–C11) benzene ring] and C—Cl⋯π inter­actions (Table 1 ▸) connect these ribbons along the [010] and [001] directions to generate a three-dimensional supra­molecular network (supplementary Figs. S5–S7).

**Figure 5:**
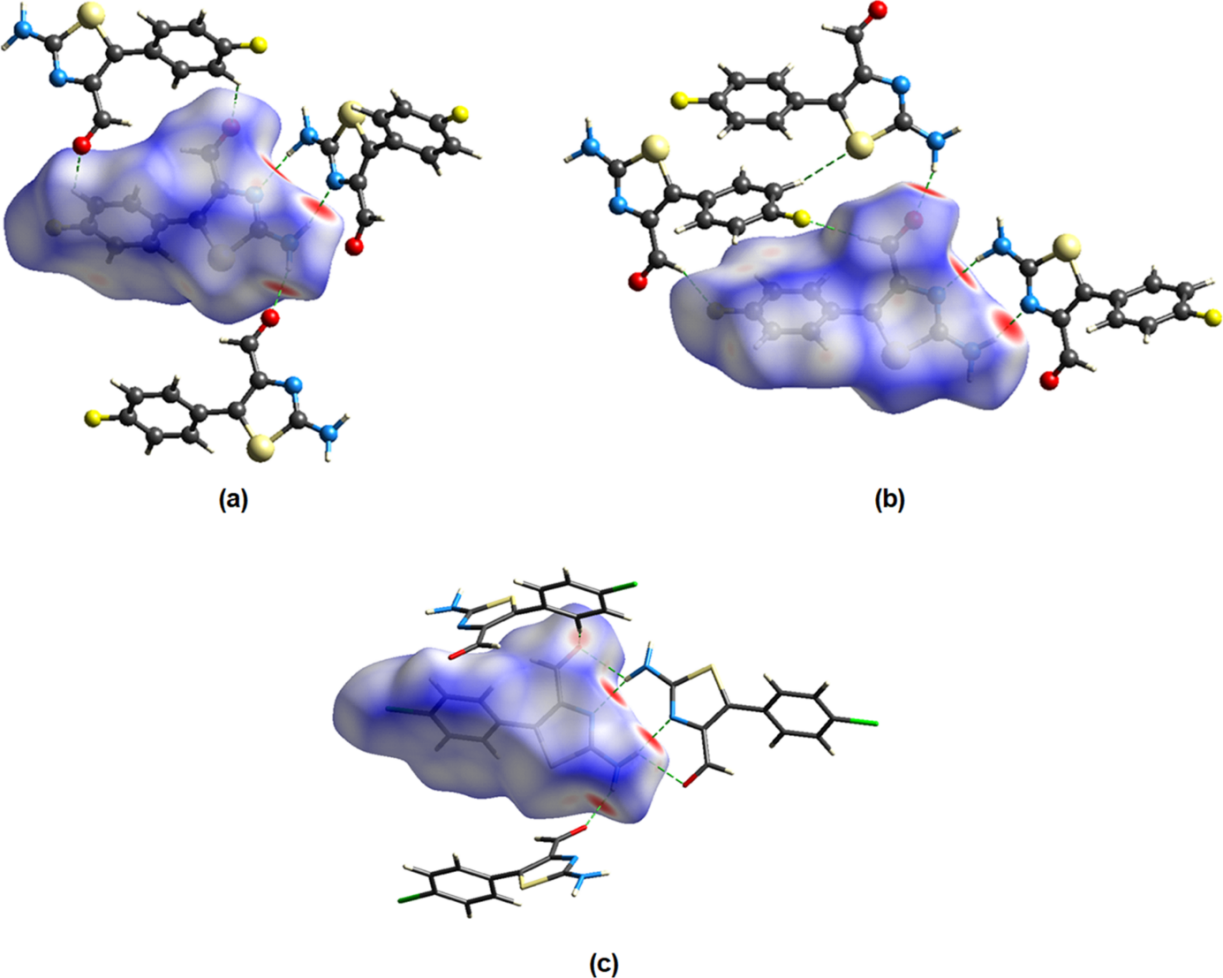
View of the three-dimensional Hirshfeld surfaces of the mol­ecules *A* (*a*) and *B* (*b*) of (I), and (*c*) (II) plotted over *d*_norm_.

*Crystal Explorer 21* (Spackman *et al.*, 2021 ▸) was used to construct Hirshfeld surfaces for both independent mol­ecules *A* and *B* in the asymmetric unit of compound (I). The *d*_norm_ mappings for mol­ecule *A* were performed in the range of −0.49 to +1.11 a.u., and for mol­ecule *B* in the range of −0.49 to +1.11 a.u. On the *d*_norm_ surfaces, bold red circles show the locations of N—H⋯O and N—H⋯N inter­actions (Fig. 5 ▸). Smaller red spots are caused by the C—H⋯S inter­actions.

**Figure 6:**
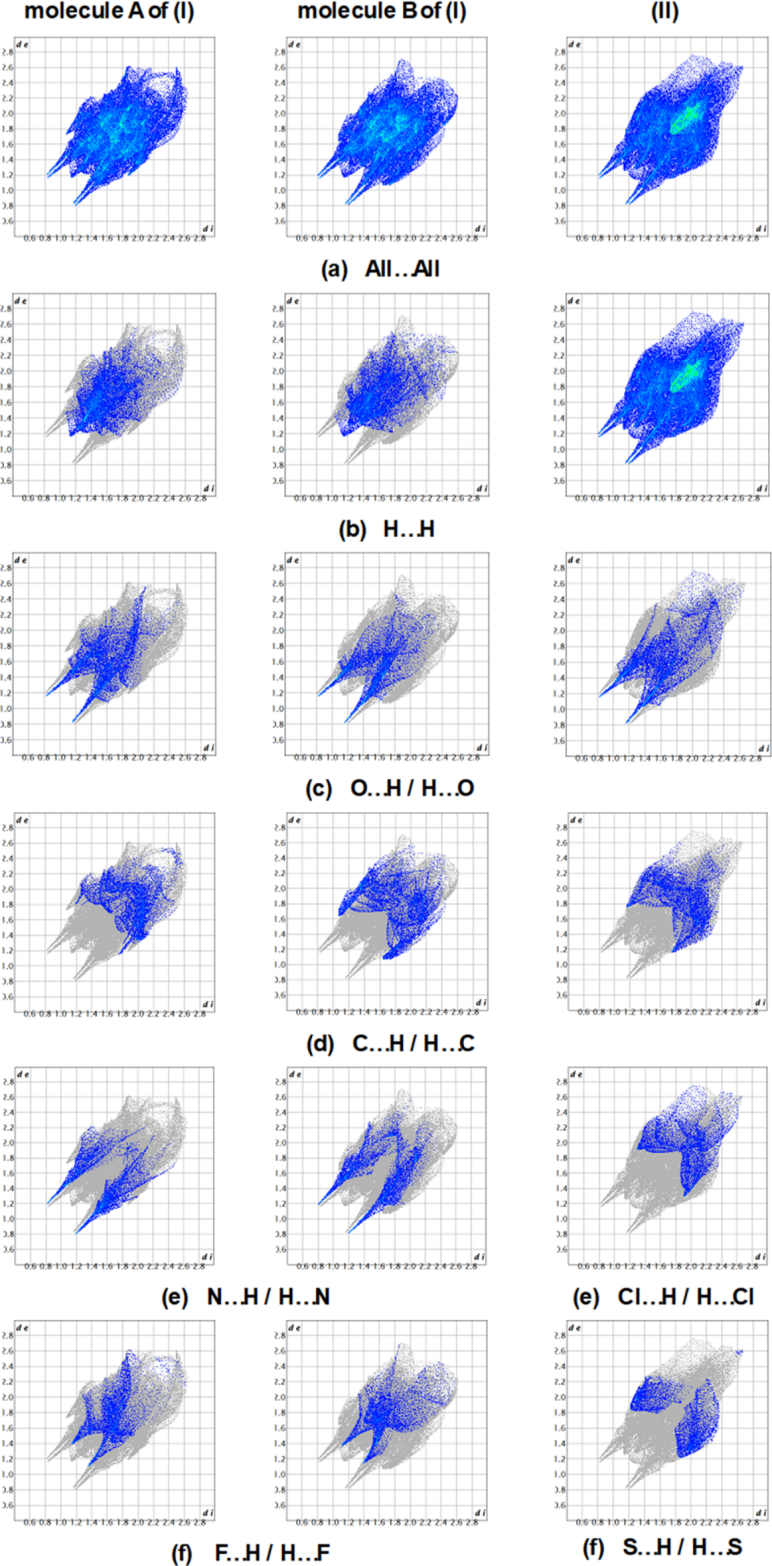
Two-dimensional fingerprint plots of mol­ecules *A* and *B* of (I), and (II) showing (*a*) all inter­actions, and delineated into (*b*) H⋯H, (*c*) O⋯H/H⋯O, (*d*) C⋯H/H⋯C, (*e*) N⋯H/H⋯N for *A* and *B* of (I) and Cl⋯H/H⋯Cl for (II), and (*f*) F⋯H/H⋯F for *A* and *B* of (I) and S⋯H/H⋯S for (II), inter­actions.

Fingerprint plots (Fig. 6 ▸) for (I) reveal that H⋯H (21.1% for mol­ecule *A* and 20.3% for mol­ecule *B*) inter­actions make the largest contributions to the surface contacts and O⋯H/H⋯O (16.0% for *A* and 13.5% for *B*), C⋯H/H⋯C (13.1% for *A* and 16.1% for *B*), N⋯H/H⋯N (11.7% for *A* and 13.1% for *B*) and F⋯H/H⋯F (10.3% for *A* and 10.2% for *B*) contacts are also significant. The inter­actions that have less of an influence include S⋯H/H⋯S (9.7% for *A* and 6.2% for *B*), C⋯C (5.6% for *A* and 5.8% for *B*), F⋯C/C⋯F (4.3% for *A* and *B*), S⋯F/F⋯S (2.6% for *A* and 3.2% for *B*), S⋯C/C⋯S (1.9% for *A* and 2.7% for *B*), F⋯O/O⋯F (1.0% for *A* and 0.9% for *B*), F⋯F (0.6% for *A* and 0.0% for *B*) and F⋯N/N⋯F (0.2% for *A* and *B*).

The solid-state consolidation in (II) is significantly impacted by H⋯H inter­actions, which account for 21.0% of the total. The inter­actions that have less of an influence include O⋯H/H⋯O (15.3%), C⋯H/H⋯C (12.1%), Cl⋯H/H⋯Cl (9.9%) and S⋯H/H⋯S (7.9%), C⋯C (6.8%), Cl⋯C/C⋯Cl (6.7%), Cl⋯S/S⋯Cl (3.7%), S⋯O / O⋯S (2.1%), Cl⋯Cl (1.8%), Cl⋯N/N⋯Cl (0.8%), N⋯C/C⋯N (0.5%), S⋯S (0.31%) and S⋯C / C⋯S (0.2%).

While the contributions of the strong inter­actions of (I) and (II) are quite consistent, weak inter­actions vary slightly depending on the mol­ecular conformation and the environment of the mol­ecules.

## 4. Database survey

The most closely related ten structures containing a 5-phenyl-1,3-thia­zole fragment are as follows: Cambridge Structural Database (CSD, Version 6.00, update of April 2025; Groom *et al.*, 2016 ▸) refcodes MEFVUS (Guseinov *et al.*, 2022 ▸), IQUHOT (Saravanan *et al.*, 2016 ▸), GUVVAW (Akkurt *et al.*, 2015 ▸), WOJKOX (Mague *et al.*, 2014 ▸), HOQSAJ (El Ashry *et al.*, 2014 ▸), SAYXEW (Sun *et al.*, 2006 ▸), EKEZUP (Rybakov *et al.*, 2003 ▸), HIYLOQ (Au-Alvarez *et al.*, 1999 ▸), FUHJIB (Caldwell *et al.*, 1987 ▸) and CPYPTZ (Le Count & Jarvis, 1977 ▸).

In the crystal of MEFVUS, C—H⋯π inter­actions link the mol­ecules, forming a three-dimensional network. In IQUHOT, the mol­ecules are linked *via* C—H⋯O inter­actions, which form *C*(7) chains propagating along [010]. In the crystal of GUVVAW, the mol­ecular packing features C—H⋯O and C—H⋯π inter­actions, forming a three-dimensional network. In WOJKOX, the two independent mol­ecules are associated *via* complementary N—H⋯N hydrogen bonds into a dimer. These dimers are associated through weak C— H⋯Cl and C—H⋯S inter­actions into supra­molecular chains propagating along the *a*-axis direction. In HOQSAJ, mol­ecular pairs connect by forming 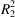
(8) motifs *via* N—H⋯N inter­actions. A three-dimensional network is established through C—H⋯π and C—Br⋯π inter­actions. In SAYXEW, similarly to HOQSAJ, mol­ecular pairs come together *via* N—H⋯N inter­actions to form 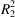
(8) motifs. A three-dimensional network is formed with C—H⋯π inter­actions. In EKEZUP, mol­ecules form extended chains through O—H⋯N hydrogen bonds. In HIYLOQ, mol­ecules are linked in parallel layers through N—H⋯N and N—H⋯S inter­actions in the *bc* plane. The layers are connected by C—H⋯π inter­actions. In FUHJIB, mol­ecules are connected to each other by forming ribbons in the [110] direction. The mol­ecular packing features C—H⋯O and C—H⋯F inter­actions. Additional C—F⋯π and C—O⋯π inter­actions consolidate the packing. In CPYPTZ, mol­ecules are linked in the *b*-axis direction as *C*(7) zigzag chains through N—H⋯N inter­actions. The mol­ecules form a three-dimensional network *via* C≡C⋯π inter­actions.

## 5. Synthesis and crystallization

**Figure 7:**
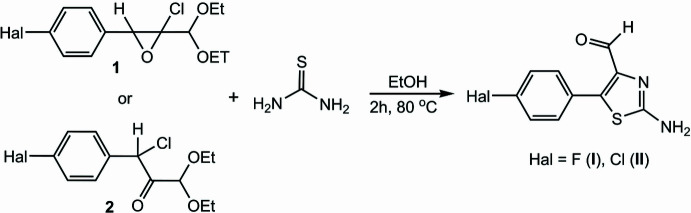
Synthesis scheme for (I) and (II).

To a solution of 2-chloro-2-(di­eth­oxy­meth­yl)-3-(4-fluoro­phen­yl)oxirane [also called 1-chloro-3,3-dieth­oxy-1-(4-fluoro­phen­yl)propan-2-one] (1.00 mmol) in 20 ml of ethanol (95%) was added thio­urea (1.00 mmol) and refluxed at 353 K for 2 h (Fig. 7 ▸). Then the ethanol was evacuated under vacuum and the resulting yellow powder of (I) was recrystallized from diethyl ether solution. Crystals suitable for X-ray diffraction were obtained by crystallization of this yellow powder from di­methyl­sulfoxide (DMSO) solution: yield: 91 or 73%; m.p. 378–380 K. Analysis calculated (%) for C_10_H_7_FN_2_OS: C 54.05, H 3.17, N 12.61; found C 54.04, H 3.15, N 12.58. ^1^H NMR (300MHz, DMSO-*d*_6_): 6.73 (2H, NH2), 7.38–7.75 (4H, Ar), 9.48 (1H, CHO). ^13^C NMR (75MHz, DMSO-*d*_6_): 116.07, 116.51, 124.00, 132.13, 132.30, 136.44, 137.62, 160.66, 165.60, 167.03, 180.12.

2-Chloro-2-(di­eth­oxy­meth­yl)-3-(4-chloro­phen­yl)oxirane [also called 1-chloro-3,3-dieth­oxy-1-(4-chloro­phen­yl)propan-2-one] was used as a starting material in the synthesis of (II), otherwise the synthetic procedure was the same as for (I): yield: 95 or 77%; m.p. 397–398 K. Analysis calculated (%) for C_10_H_7_ClN_2_OS: C 50.32, H 2.96, N 11.74; found C 50.28 H 2.95, N 11.70. ^1^H NMR (300MHz, DMSO-*d*_6_): 7.51–7.66 (4H, Ar), 8.36 (2H, NH2), 9.48 (1H, CHO). ^13^C NMR (75MHz, DMSO-*d*_6_): 126.18, 129.42, 131.67, 135.08, 135.49, 136.94, 167.45, 179.75.

## 6. Refinement

Crystal data, data collection and structure refinement details are summarized in Table 3 ▸. The C-bound H atoms were positioned geometrically and refined using a riding model, with C—H = 0.95 Å. The H atoms of the NH_2_ groups were found in difference-Fourier maps and their positions were freely refined.

## Supplementary Material

Crystal structure: contains datablock(s) I, II. DOI: 10.1107/S2056989025010667/hb8159sup1.cif

Structure factors: contains datablock(s) I. DOI: 10.1107/S2056989025010667/hb8159Isup4.hkl

Structure factors: contains datablock(s) II. DOI: 10.1107/S2056989025010667/hb8159IIsup5.hkl

Supporting information file. DOI: 10.1107/S2056989025010667/hb8159Isup4.cml

Supporting information file. DOI: 10.1107/S2056989025010667/hb8159IIsup5.cml

Supplementary Material. DOI: 10.1107/S2056989025010667/hb8159sup6.pdf

CCDC references: 2512074, 2512073

Additional supporting information:  crystallographic information; 3D view; checkCIF report

## Figures and Tables

**Table 1:** Hydrogen-bond geometry (Å, °) for (I)

*D*—H⋯*A*	*D*—H	H⋯*A*	*D*⋯*A*	*D*—H⋯*A*
N12*A*—H12*A*⋯N3*B*	0.87 (2)	2.16 (3)	3.0121 (19)	167 (2)
N12*A*—H12*B*⋯O14*A*^i^	0.86 (2)	2.13 (2)	2.9596 (19)	160 (2)
N12*B*—H12*C*⋯O14*B*^ii^	0.82 (2)	2.16 (2)	2.9433 (19)	161 (2)
N12*B*—H12*D*⋯N3*A*	0.87 (3)	2.13 (3)	2.9778 (19)	164 (2)
C8*A*—H8*A*⋯S1*B*^iii^	0.95	2.91	3.6376 (16)	134
C8*B*—H8*B*⋯N3*A*^iv^	0.95	2.68	3.521 (2)	149
C13*B*—H13*B*⋯F9*A*^v^	0.95	2.60	3.4911 (19)	157
C7*B*—H7*B*⋯*Cg*3^iv^	0.95	2.75	3.4117 (17)	127

Symmetry codes: (i) 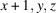; (ii) 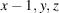; (iii) 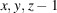; (iv) 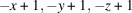; (v) 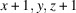.

**Table 2:** Hydrogen-bond geometry (Å, °) for (II)

*D*—H⋯*A*	*D*—H	H⋯*A*	*D*⋯*A*	*D*—H⋯*A*
N12—H12*A*⋯N3^i^	0.84 (3)	2.19 (3)	3.015 (2)	167 (2)
N12—H12*A*⋯O14^i^	0.84 (3)	2.61 (3)	3.1222 (19)	121 (2)
N12—H12*B*⋯O14^ii^	0.81 (3)	2.18 (3)	2.940 (2)	159 (2)
C9—Cl9⋯*Cg*1^iii^	1.74 (1)	3.53 (1)	4.5317 (19)	114 (1)

Symmetry codes: (i) 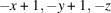; (ii) 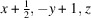; (iii) 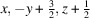.

**Table 3:** Experimental details

	(I)	(II)
Crystal data		
Chemical formula	C_10_H_7_FN_2_OS	C_10_H_7_ClN_2_OS
*M*_r_	222.24	238.69
Crystal system, space group	Triclinic, *P* 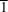	Monoclinic, *I*2/*a*
Temperature (K)	100	100
*a*, *b*, *c* (Å)	7.6272 (1), 9.0292 (1), 14.7403 (3)	13.9857 (2), 9.8459 (1), 15.3349 (2)
α, β, γ (°)	90.567 (1), 98.122 (1), 108.526 (1)	90, 105.170 (1), 90
*V* (Å^3^)	951.24 (3)	2038.06 (5)
*Z*	4	8
Radiation type	Cu *K*α	Cu *K*α
μ (mm^−1^)	2.95	5.01
Crystal size (mm)	0.27 × 0.22 × 0.15	0.53 × 0.38 × 0.30

Data collection		
Diffractometer	XtaLAB Synergy, Dualflex, HyPix	XtaLAB Synergy, Dualflex, HyPix
Absorption correction	Gaussian (*CrysAlis PRO*; Rigaku OD, 2025 ▸)	Gaussian (CrysAlisPr; (Rigaku OD, 2025 ▸)
*T*_min_, *T*_max_	0.482, 0.642	0.169, 0.859
No. of measured, independent and observed [*I* > 2σ(*I*)] reflections	25299, 4097, 3888	13963, 2220, 2165
*R*_int_	0.047	0.043
(sin θ/λ)_max_ (Å^−1^)	0.638	0.639

Refinement		
*R*[*F*^2^ > 2σ(*F*^2^)], *wR*(*F*^2^), *S*	0.036, 0.098, 1.09	0.036, 0.094, 1.07
No. of reflections	4097	2220
No. of parameters	287	144
H-atom treatment	H atoms treated by a mixture of independent and constrained refinement	H atoms treated by a mixture of independent and constrained refinement
Δρ_max_, Δρ_min_ (e Å^−3^)	0.30, −0.32	0.37, −0.37

Computer programs: *CrysAlis PRO* (Rigaku OD, 2025 ▸), *SHELXS97* (Sheldrick, 2008 ▸), *SHELXL2014* (Sheldrick, 2015 ▸), *ORTEP-3 for Windows* (Farrugia, 2012 ▸) and *PLATON* (Spek, 2020 ▸).

## supplementary crystallographic information

### 2-Amino-5-(4-fluorophenyl)-1,3-thiazole-4-carbaldehyde (I) Crystal data

**Table:**

C_10_H_7_FN_2_OS	*Z* = 4
*M**_r_* = 222.24	*F*(000) = 456
Triclinic, *P*1	*D*_x_ = 1.552 Mg m^−^^3^
*a* = 7.6272 (1) Å	Cu *K*α radiation, λ = 1.54184 Å
*b* = 9.0292 (1) Å	Cell parameters from 15352 reflections
*c* = 14.7403 (3) Å	θ = 3.0–79.1°
α = 90.567 (1)°	µ = 2.95 mm^−^^1^
β = 98.122 (1)°	*T* = 100 K
γ = 108.526 (1)°	Prism, yellow
*V* = 951.24 (3) Å^3^	0.27 × 0.22 × 0.15 mm

### 2-Amino-5-(4-fluorophenyl)-1,3-thiazole-4-carbaldehyde (I) Data collection

**Table:**

XtaLAB Synergy, Dualflex, HyPix diffractometer	4097 independent reflections
Radiation source: micro-focus sealed X-ray tube, PhotonJet (Cu) X-ray Source	3888 reflections with *I* > 2σ(*I*)
Mirror monochromator	*R*_int_ = 0.047
Detector resolution: 10.0000 pixels mm^-1^	θ_max_ = 79.8°, θ_min_ = 3.0°
ω scans	*h* = −9→9
Absorption correction: gaussian (CrysAlisPro; Rigaku OD, 2025)	*k* = −11→11
*T*_min_ = 0.482, *T*_max_ = 0.642	*l* = −18→18
25299 measured reflections	

### 2-Amino-5-(4-fluorophenyl)-1,3-thiazole-4-carbaldehyde (I) Refinement

**Table:**

Refinement on *F*^2^	Primary atom site location: structure-invariant direct methods
Least-squares matrix: full	Secondary atom site location: difference Fourier map
*R*[*F*^2^ > 2σ(*F*^2^)] = 0.036	Hydrogen site location: mixed
*wR*(*F*^2^) = 0.098	H atoms treated by a mixture of independent and constrained refinement
*S* = 1.09	*w* = 1/[σ^2^(*F*_o_^2^) + (0.0488*P*)^2^ + 0.4701*P*] where *P* = (*F*_o_^2^ + 2*F*_c_^2^)/3
4097 reflections	(Δ/σ)_max_ = 0.001
287 parameters	Δρ_max_ = 0.30 e Å^−^^3^
0 restraints	Δρ_min_ = −0.32 e Å^−^^3^

### 2-Amino-5-(4-fluorophenyl)-1,3-thiazole-4-carbaldehyde (I) Special details

**Table:**

Geometry. All esds (except the esd in the dihedral angle between two l.s. planes) are estimated using the full covariance matrix. The cell esds are taken into account individually in the estimation of esds in distances, angles and torsion angles; correlations between esds in cell parameters are only used when they are defined by crystal symmetry. An approximate (isotropic) treatment of cell esds is used for estimating esds involving l.s. planes.

### 2-Amino-5-(4-fluorophenyl)-1,3-thiazole-4-carbaldehyde (I) Fractional atomic coordinates and isotropic or equivalent isotropic displacement parameters (Å^2^)

**Table:**

	*x*	*y*	*z*	*U*_iso_*/*U*_eq_	
C2A	0.6615 (2)	0.83196 (18)	0.12921 (10)	0.0221 (3)	
C2B	0.4924 (2)	0.83630 (18)	0.38518 (10)	0.0213 (3)	
C4A	0.3723 (2)	0.77903 (18)	0.05708 (10)	0.0220 (3)	
C4B	0.7501 (2)	0.80308 (18)	0.45848 (10)	0.0219 (3)	
C5A	0.4497 (2)	0.75647 (19)	−0.01828 (11)	0.0229 (3)	
C5B	0.6445 (2)	0.76230 (18)	0.52804 (10)	0.0215 (3)	
C6A	0.3598 (2)	0.70487 (19)	−0.11352 (10)	0.0232 (3)	
C6B	0.6890 (2)	0.71135 (18)	0.62088 (10)	0.0214 (3)	
C7A	0.4107 (2)	0.80140 (19)	−0.18549 (11)	0.0257 (3)	
H7A	0.507656	0.899145	−0.173195	0.031*	
C7B	0.5773 (2)	0.56850 (19)	0.64774 (11)	0.0238 (3)	
H7B	0.474834	0.504211	0.605476	0.029*	
C8A	0.3209 (2)	0.7556 (2)	−0.27469 (11)	0.0274 (3)	
H8A	0.352042	0.822314	−0.323469	0.033*	
C8B	0.6136 (2)	0.51914 (19)	0.73517 (11)	0.0245 (3)	
H8B	0.538805	0.421354	0.753172	0.029*	
C9A	0.1857 (2)	0.6112 (2)	−0.29078 (11)	0.0271 (3)	
C9B	0.7615 (2)	0.6165 (2)	0.79501 (10)	0.0236 (3)	
C10A	0.1335 (2)	0.5107 (2)	−0.22235 (12)	0.0276 (3)	
H10A	0.040183	0.411258	−0.235969	0.033*	
C10B	0.8742 (2)	0.7586 (2)	0.77200 (11)	0.0261 (3)	
H10B	0.974704	0.822805	0.815248	0.031*	
C11A	0.2213 (2)	0.5591 (2)	−0.13282 (11)	0.0252 (3)	
H11A	0.186923	0.492471	−0.084345	0.030*	
C11B	0.8374 (2)	0.80602 (19)	0.68379 (11)	0.0241 (3)	
H11B	0.913848	0.903551	0.666320	0.029*	
C13A	0.1778 (2)	0.7715 (2)	0.05284 (11)	0.0256 (3)	
H13A	0.097536	0.741770	−0.004340	0.031*	
C13B	0.9386 (2)	0.79426 (19)	0.46269 (11)	0.0244 (3)	
H13B	0.993238	0.760225	0.517060	0.029*	
F9A	0.09835 (16)	0.56466 (13)	−0.37805 (7)	0.0361 (3)	
F9B	0.79817 (14)	0.56898 (12)	0.88103 (6)	0.0293 (2)	
N3A	0.49060 (18)	0.82060 (15)	0.14004 (9)	0.0215 (3)	
N3B	0.66485 (18)	0.84586 (16)	0.37814 (9)	0.0216 (3)	
N12A	0.8086 (2)	0.87201 (17)	0.19689 (10)	0.0257 (3)	
N12B	0.3726 (2)	0.86969 (18)	0.31901 (9)	0.0247 (3)	
O14A	0.11253 (16)	0.80146 (16)	0.11929 (8)	0.0305 (3)	
O14B	1.02965 (16)	0.82851 (15)	0.39967 (8)	0.0294 (3)	
S1A	0.68838 (5)	0.79416 (5)	0.01534 (3)	0.02509 (11)	
S1B	0.42376 (5)	0.77503 (5)	0.49132 (2)	0.02230 (11)	
H12A	0.786 (3)	0.873 (3)	0.2530 (17)	0.036 (6)*	
H12B	0.911 (3)	0.856 (2)	0.1879 (15)	0.029 (5)*	
H12C	0.265 (3)	0.853 (2)	0.3284 (15)	0.028 (5)*	
H12D	0.392 (3)	0.865 (3)	0.2623 (19)	0.043 (6)*	

### 2-Amino-5-(4-fluorophenyl)-1,3-thiazole-4-carbaldehyde (I) Atomic displacement parameters (Å^2^)

**Table:**

	*U*^11^	*U*^22^	*U*^33^	*U*^12^	*U*^13^	*U*^23^
C2A	0.0243 (7)	0.0251 (7)	0.0184 (7)	0.0090 (6)	0.0054 (6)	0.0024 (6)
C2B	0.0240 (7)	0.0248 (7)	0.0153 (7)	0.0081 (6)	0.0033 (5)	0.0016 (5)
C4A	0.0232 (7)	0.0257 (7)	0.0172 (7)	0.0078 (6)	0.0038 (6)	0.0026 (6)
C4B	0.0229 (7)	0.0248 (7)	0.0166 (7)	0.0064 (6)	0.0013 (6)	0.0018 (5)
C5A	0.0251 (7)	0.0254 (7)	0.0198 (7)	0.0094 (6)	0.0055 (6)	0.0040 (6)
C5B	0.0216 (7)	0.0242 (7)	0.0176 (7)	0.0066 (6)	0.0011 (6)	0.0000 (6)
C6A	0.0270 (7)	0.0293 (8)	0.0171 (7)	0.0137 (6)	0.0054 (6)	0.0032 (6)
C6B	0.0231 (7)	0.0272 (8)	0.0155 (7)	0.0107 (6)	0.0028 (5)	0.0017 (6)
C7A	0.0311 (8)	0.0274 (8)	0.0207 (8)	0.0110 (6)	0.0072 (6)	0.0020 (6)
C7B	0.0248 (7)	0.0266 (8)	0.0194 (7)	0.0084 (6)	0.0012 (6)	−0.0020 (6)
C8A	0.0363 (9)	0.0327 (8)	0.0183 (7)	0.0159 (7)	0.0092 (6)	0.0065 (6)
C8B	0.0288 (8)	0.0253 (7)	0.0205 (7)	0.0094 (6)	0.0058 (6)	0.0041 (6)
C9A	0.0321 (8)	0.0370 (9)	0.0157 (7)	0.0175 (7)	0.0007 (6)	−0.0003 (6)
C9B	0.0291 (8)	0.0319 (8)	0.0136 (7)	0.0153 (6)	0.0034 (6)	0.0037 (6)
C10A	0.0285 (8)	0.0303 (8)	0.0235 (8)	0.0099 (7)	0.0019 (6)	0.0014 (6)
C10B	0.0259 (8)	0.0323 (8)	0.0190 (7)	0.0095 (6)	−0.0011 (6)	0.0000 (6)
C11A	0.0277 (8)	0.0298 (8)	0.0197 (7)	0.0110 (6)	0.0046 (6)	0.0055 (6)
C11B	0.0236 (7)	0.0273 (8)	0.0202 (7)	0.0070 (6)	0.0020 (6)	0.0031 (6)
C13A	0.0220 (7)	0.0342 (8)	0.0200 (7)	0.0083 (6)	0.0024 (6)	0.0016 (6)
C13B	0.0223 (7)	0.0284 (8)	0.0215 (7)	0.0074 (6)	0.0019 (6)	0.0033 (6)
F9A	0.0448 (6)	0.0443 (6)	0.0173 (5)	0.0153 (5)	−0.0030 (4)	−0.0012 (4)
F9B	0.0375 (5)	0.0352 (5)	0.0157 (4)	0.0133 (4)	0.0017 (4)	0.0060 (4)
N3A	0.0219 (6)	0.0266 (6)	0.0168 (6)	0.0082 (5)	0.0039 (5)	0.0024 (5)
N3B	0.0221 (6)	0.0272 (6)	0.0157 (6)	0.0082 (5)	0.0027 (5)	0.0016 (5)
N12A	0.0219 (7)	0.0369 (8)	0.0191 (7)	0.0109 (6)	0.0032 (5)	−0.0011 (5)
N12B	0.0227 (7)	0.0383 (8)	0.0167 (6)	0.0140 (6)	0.0048 (5)	0.0051 (5)
O14A	0.0261 (6)	0.0447 (7)	0.0237 (6)	0.0140 (5)	0.0077 (5)	0.0019 (5)
O14B	0.0244 (6)	0.0378 (7)	0.0282 (6)	0.0109 (5)	0.0087 (5)	0.0069 (5)
S1A	0.0238 (2)	0.0362 (2)	0.01781 (19)	0.01206 (16)	0.00570 (14)	0.00075 (15)
S1B	0.02187 (19)	0.0323 (2)	0.01454 (18)	0.01058 (15)	0.00423 (13)	0.00355 (14)

### 2-Amino-5-(4-fluorophenyl)-1,3-thiazole-4-carbaldehyde (I) Geometric parameters (Å, º)

**Table:**

C2A—N3A	1.308 (2)	C7B—H7B	0.9500
C2A—N12A	1.343 (2)	C8A—C9A	1.375 (3)
C2A—S1A	1.7623 (16)	C8A—H8A	0.9500
C2B—N3B	1.309 (2)	C8B—C9B	1.377 (2)
C2B—N12B	1.344 (2)	C8B—H8B	0.9500
C2B—S1B	1.7585 (15)	C9A—F9A	1.3580 (18)
C4A—C5A	1.372 (2)	C9A—C10A	1.379 (2)
C4A—N3A	1.384 (2)	C9B—F9B	1.3626 (17)
C4A—C13A	1.455 (2)	C9B—C10B	1.375 (2)
C4B—C5B	1.374 (2)	C10A—C11A	1.391 (2)
C4B—N3B	1.388 (2)	C10A—H10A	0.9500
C4B—C13B	1.458 (2)	C10B—C11B	1.392 (2)
C5A—C6A	1.473 (2)	C10B—H10B	0.9500
C5A—S1A	1.7391 (16)	C11A—H11A	0.9500
C5B—C6B	1.477 (2)	C11B—H11B	0.9500
C5B—S1B	1.7342 (16)	C13A—O14A	1.224 (2)
C6A—C11A	1.397 (2)	C13A—H13A	0.9500
C6A—C7A	1.399 (2)	C13B—O14B	1.222 (2)
C6B—C11B	1.394 (2)	C13B—H13B	0.9500
C6B—C7B	1.398 (2)	N12A—H12A	0.87 (2)
C7A—C8A	1.387 (2)	N12A—H12B	0.86 (2)
C7A—H7A	0.9500	N12B—H12C	0.82 (2)
C7B—C8B	1.388 (2)	N12B—H12D	0.87 (3)
			
N3A—C2A—N12A	124.52 (14)	C7B—C8B—H8B	121.1
N3A—C2A—S1A	114.34 (12)	F9A—C9A—C8A	118.78 (15)
N12A—C2A—S1A	121.12 (12)	F9A—C9A—C10A	118.06 (16)
N3B—C2B—N12B	124.89 (14)	C8A—C9A—C10A	123.16 (15)
N3B—C2B—S1B	114.47 (11)	F9B—C9B—C10B	118.52 (14)
N12B—C2B—S1B	120.64 (12)	F9B—C9B—C8B	118.27 (14)
C5A—C4A—N3A	117.22 (14)	C10B—C9B—C8B	123.21 (14)
C5A—C4A—C13A	123.57 (14)	C9A—C10A—C11A	118.06 (16)
N3A—C4A—C13A	119.09 (13)	C9A—C10A—H10A	121.0
C5B—C4B—N3B	116.79 (14)	C11A—C10A—H10A	121.0
C5B—C4B—C13B	123.91 (14)	C9B—C10B—C11B	118.36 (15)
N3B—C4B—C13B	119.19 (13)	C9B—C10B—H10B	120.8
C4A—C5A—C6A	129.84 (15)	C11B—C10B—H10B	120.8
C4A—C5A—S1A	108.53 (12)	C10A—C11A—C6A	120.60 (15)
C6A—C5A—S1A	121.61 (12)	C10A—C11A—H11A	119.7
C4B—C5B—C6B	131.24 (14)	C6A—C11A—H11A	119.7
C4B—C5B—S1B	108.86 (11)	C10B—C11B—C6B	120.44 (15)
C6B—C5B—S1B	119.89 (11)	C10B—C11B—H11B	119.8
C11A—C6A—C7A	119.25 (15)	C6B—C11B—H11B	119.8
C11A—C6A—C5A	120.25 (14)	O14A—C13A—C4A	123.26 (15)
C7A—C6A—C5A	120.50 (15)	O14A—C13A—H13A	118.4
C11B—C6B—C7B	119.10 (14)	C4A—C13A—H13A	118.4
C11B—C6B—C5B	121.07 (14)	O14B—C13B—C4B	123.17 (15)
C7B—C6B—C5B	119.79 (14)	O14B—C13B—H13B	118.4
C8A—C7A—C6A	120.52 (16)	C4B—C13B—H13B	118.4
C8A—C7A—H7A	119.7	C2A—N3A—C4A	110.35 (13)
C6A—C7A—H7A	119.7	C2B—N3B—C4B	110.30 (13)
C8B—C7B—C6B	121.03 (15)	C2A—N12A—H12A	117.5 (16)
C8B—C7B—H7B	119.5	C2A—N12A—H12B	119.2 (14)
C6B—C7B—H7B	119.5	H12A—N12A—H12B	118 (2)
C9A—C8A—C7A	118.37 (15)	C2B—N12B—H12C	117.4 (15)
C9A—C8A—H8A	120.8	C2B—N12B—H12D	118.1 (17)
C7A—C8A—H8A	120.8	H12C—N12B—H12D	118 (2)
C9B—C8B—C7B	117.85 (15)	C5A—S1A—C2A	89.53 (7)
C9B—C8B—H8B	121.1	C5B—S1B—C2B	89.59 (7)
			
N3A—C4A—C5A—C6A	176.64 (15)	F9B—C9B—C10B—C11B	179.38 (14)
C13A—C4A—C5A—C6A	−7.5 (3)	C8B—C9B—C10B—C11B	−0.3 (3)
N3A—C4A—C5A—S1A	−1.54 (18)	C9A—C10A—C11A—C6A	−0.7 (2)
C13A—C4A—C5A—S1A	174.34 (13)	C7A—C6A—C11A—C10A	−0.6 (2)
N3B—C4B—C5B—C6B	179.83 (15)	C5A—C6A—C11A—C10A	178.85 (15)
C13B—C4B—C5B—C6B	3.8 (3)	C9B—C10B—C11B—C6B	0.3 (2)
N3B—C4B—C5B—S1B	0.85 (18)	C7B—C6B—C11B—C10B	0.3 (2)
C13B—C4B—C5B—S1B	−175.17 (13)	C5B—C6B—C11B—C10B	178.03 (15)
C4A—C5A—C6A—C11A	−60.0 (2)	C5A—C4A—C13A—O14A	−175.19 (17)
S1A—C5A—C6A—C11A	118.02 (15)	N3A—C4A—C13A—O14A	0.6 (3)
C4A—C5A—C6A—C7A	119.5 (2)	C5B—C4B—C13B—O14B	178.76 (16)
S1A—C5A—C6A—C7A	−62.50 (19)	N3B—C4B—C13B—O14B	2.8 (2)
C4B—C5B—C6B—C11B	58.8 (2)	N12A—C2A—N3A—C4A	179.29 (15)
S1B—C5B—C6B—C11B	−122.33 (15)	S1A—C2A—N3A—C4A	0.69 (17)
C4B—C5B—C6B—C7B	−123.49 (19)	C5A—C4A—N3A—C2A	0.6 (2)
S1B—C5B—C6B—C7B	55.40 (19)	C13A—C4A—N3A—C2A	−175.49 (14)
C11A—C6A—C7A—C8A	2.1 (2)	N12B—C2B—N3B—C4B	−179.90 (15)
C5A—C6A—C7A—C8A	−177.39 (15)	S1B—C2B—N3B—C4B	0.31 (17)
C11B—C6B—C7B—C8B	−0.9 (2)	C5B—C4B—N3B—C2B	−0.8 (2)
C5B—C6B—C7B—C8B	−178.70 (14)	C13B—C4B—N3B—C2B	175.45 (14)
C6A—C7A—C8A—C9A	−2.2 (2)	C4A—C5A—S1A—C2A	1.52 (12)
C6B—C7B—C8B—C9B	1.0 (2)	C6A—C5A—S1A—C2A	−176.85 (14)
C7A—C8A—C9A—F9A	−179.31 (15)	N3A—C2A—S1A—C5A	−1.32 (13)
C7A—C8A—C9A—C10A	0.8 (3)	N12A—C2A—S1A—C5A	−179.98 (14)
C7B—C8B—C9B—F9B	180.00 (14)	C4B—C5B—S1B—C2B	−0.53 (12)
C7B—C8B—C9B—C10B	−0.4 (2)	C6B—C5B—S1B—C2B	−179.65 (13)
F9A—C9A—C10A—C11A	−179.26 (14)	N3B—C2B—S1B—C5B	0.13 (13)
C8A—C9A—C10A—C11A	0.6 (3)	N12B—C2B—S1B—C5B	−179.67 (14)

### 2-Amino-5-(4-fluorophenyl)-1,3-thiazole-4-carbaldehyde (I) Hydrogen-bond geometry (Å, º)

**Table:**

*D*—H···*A*	*D*—H	H···*A*	*D*···*A*	*D*—H···*A*
N12*A*—H12*A*···N3*B*	0.87 (2)	2.16 (3)	3.0121 (19)	167 (2)
N12*A*—H12*B*···O14*A*^i^	0.86 (2)	2.13 (2)	2.9596 (19)	160 (2)
N12*B*—H12*C*···O14*B*^ii^	0.82 (2)	2.16 (2)	2.9433 (19)	161 (2)
N12*B*—H12*D*···N3*A*	0.87 (3)	2.13 (3)	2.9778 (19)	164 (2)
C8*A*—H8*A*···S1*B*^iii^	0.95	2.91	3.6376 (16)	134
C8*B*—H8*B*···N3*A*^iv^	0.95	2.68	3.521 (2)	149
C13*B*—H13*B*···F9*A*^v^	0.95	2.60	3.4911 (19)	157
C7*B*—H7*B*···*Cg*3^iv^	0.95	2.75	3.4117 (17)	127

Symmetry codes: (i) *x*+1, *y*, *z*; (ii) *x*−1, *y*, *z*; (iii) *x*, *y*, *z*−1; (iv) −*x*+1, −*y*+1, −*z*+1; (v) *x*+1, *y*, *z*+1.

### 2-Amino-5-(4-chlorophenyl)-1,3-thiazole-4-carbaldehyde (II) Crystal data

**Table:**

C_10_H_7_ClN_2_OS	*F*(000) = 976
*M**_r_* = 238.69	*D*_x_ = 1.556 Mg m^−^^3^
Monoclinic, *I*2/*a*	Cu *K*α radiation, λ = 1.54184 Å
*a* = 13.9857 (2) Å	Cell parameters from 9428 reflections
*b* = 9.8459 (1) Å	θ = 5.4–79.9°
*c* = 15.3349 (2) Å	µ = 5.01 mm^−^^1^
β = 105.170 (1)°	*T* = 100 K
*V* = 2038.06 (5) Å^3^	Prism, yellow
*Z* = 8	0.53 × 0.38 × 0.30 mm

### 2-Amino-5-(4-chlorophenyl)-1,3-thiazole-4-carbaldehyde (II) Data collection

**Table:**

XtaLAB Synergy, Dualflex, HyPix diffractometer	2220 independent reflections
Radiation source: micro-focus sealed X-ray tube, PhotonJet (Cu) X-ray Source	2165 reflections with *I* > 2σ(*I*)
Mirror monochromator	*R*_int_ = 0.043
Detector resolution: 10.0000 pixels mm^-1^	θ_max_ = 80.3°, θ_min_ = 5.4°
ω scans	*h* = −17→17
Absorption correction: gaussian (CrysAlisPr; (Rigaku OD, 2025)	*k* = −10→12
*T*_min_ = 0.169, *T*_max_ = 0.859	*l* = −19→19
13963 measured reflections	

### 2-Amino-5-(4-chlorophenyl)-1,3-thiazole-4-carbaldehyde (II) Refinement

**Table:**

Refinement on *F*^2^	Primary atom site location: structure-invariant direct methods
Least-squares matrix: full	Secondary atom site location: difference Fourier map
*R*[*F*^2^ > 2σ(*F*^2^)] = 0.036	Hydrogen site location: mixed
*wR*(*F*^2^) = 0.094	H atoms treated by a mixture of independent and constrained refinement
*S* = 1.07	*w* = 1/[σ^2^(*F*_o_^2^) + (0.0543*P*)^2^ + 2.2643*P*] where *P* = (*F*_o_^2^ + 2*F*_c_^2^)/3
2220 reflections	(Δ/σ)_max_ = 0.001
144 parameters	Δρ_max_ = 0.37 e Å^−^^3^
0 restraints	Δρ_min_ = −0.37 e Å^−^^3^

### 2-Amino-5-(4-chlorophenyl)-1,3-thiazole-4-carbaldehyde (II) Special details

**Table:**

Geometry. All esds (except the esd in the dihedral angle between two l.s. planes) are estimated using the full covariance matrix. The cell esds are taken into account individually in the estimation of esds in distances, angles and torsion angles; correlations between esds in cell parameters are only used when they are defined by crystal symmetry. An approximate (isotropic) treatment of cell esds is used for estimating esds involving l.s. planes.

### 2-Amino-5-(4-chlorophenyl)-1,3-thiazole-4-carbaldehyde (II) Fractional atomic coordinates and isotropic or equivalent isotropic displacement parameters (Å^2^)

**Table:**

	*x*	*y*	*z*	*U*_iso_*/*U*_eq_	
C2	0.59720 (12)	0.44375 (16)	0.12413 (11)	0.0197 (3)	
C4	0.50163 (12)	0.54350 (17)	0.19941 (10)	0.0194 (3)	
C5	0.57830 (12)	0.51273 (16)	0.27300 (10)	0.0194 (3)	
C6	0.59306 (12)	0.55034 (17)	0.36882 (11)	0.0202 (3)	
C7	0.61451 (13)	0.45314 (18)	0.43726 (11)	0.0227 (3)	
H7	0.618549	0.360040	0.422360	0.027*	
C8	0.63007 (13)	0.49141 (18)	0.52726 (11)	0.0237 (3)	
H8	0.643953	0.425002	0.573823	0.028*	
C9	0.62502 (13)	0.62773 (18)	0.54796 (11)	0.0230 (3)	
C10	0.60396 (13)	0.72642 (18)	0.48120 (12)	0.0258 (4)	
H10	0.600421	0.819419	0.496565	0.031*	
C11	0.58812 (13)	0.68741 (18)	0.39163 (12)	0.0242 (4)	
H11	0.573797	0.754261	0.345349	0.029*	
C13	0.41003 (13)	0.60683 (17)	0.20523 (11)	0.0211 (3)	
H13	0.401977	0.627562	0.263401	0.025*	
Cl9	0.64554 (3)	0.67747 (4)	0.66029 (3)	0.02945 (14)	
N3	0.51246 (10)	0.50539 (14)	0.11549 (9)	0.0193 (3)	
N12	0.62848 (12)	0.39102 (17)	0.05542 (10)	0.0240 (3)	
O14	0.34230 (9)	0.63509 (13)	0.13888 (8)	0.0242 (3)	
S1	0.66956 (3)	0.42813 (4)	0.23650 (2)	0.02012 (13)	
H12A	0.597 (2)	0.418 (3)	0.0043 (19)	0.033 (6)*	
H12B	0.687 (2)	0.376 (2)	0.0650 (17)	0.028 (6)*	

### 2-Amino-5-(4-chlorophenyl)-1,3-thiazole-4-carbaldehyde (II) Atomic displacement parameters (Å^2^)

**Table:**

	*U*^11^	*U*^22^	*U*^33^	*U*^12^	*U*^13^	*U*^23^
C2	0.0236 (8)	0.0222 (7)	0.0133 (7)	−0.0032 (6)	0.0052 (6)	−0.0007 (6)
C4	0.0238 (8)	0.0223 (7)	0.0128 (7)	−0.0026 (6)	0.0058 (6)	−0.0007 (6)
C5	0.0224 (7)	0.0225 (7)	0.0143 (7)	0.0001 (6)	0.0065 (6)	0.0005 (6)
C6	0.0216 (7)	0.0264 (8)	0.0131 (7)	0.0009 (6)	0.0051 (6)	−0.0016 (6)
C7	0.0261 (8)	0.0248 (8)	0.0167 (8)	0.0035 (6)	0.0050 (6)	−0.0014 (6)
C8	0.0286 (8)	0.0278 (8)	0.0138 (7)	0.0052 (6)	0.0042 (6)	0.0022 (6)
C9	0.0252 (8)	0.0310 (9)	0.0123 (7)	0.0048 (6)	0.0040 (6)	−0.0034 (6)
C10	0.0333 (9)	0.0244 (8)	0.0185 (8)	0.0029 (7)	0.0048 (7)	−0.0024 (6)
C11	0.0308 (9)	0.0249 (8)	0.0157 (8)	0.0019 (6)	0.0043 (6)	0.0020 (6)
C13	0.0266 (8)	0.0226 (7)	0.0152 (7)	−0.0011 (6)	0.0075 (6)	0.0009 (6)
Cl9	0.0417 (3)	0.0328 (2)	0.0120 (2)	0.00928 (17)	0.00381 (17)	−0.00327 (14)
N3	0.0225 (6)	0.0242 (6)	0.0118 (6)	−0.0025 (5)	0.0057 (5)	−0.0015 (5)
N12	0.0228 (7)	0.0350 (8)	0.0141 (7)	0.0030 (6)	0.0047 (6)	−0.0028 (6)
O14	0.0226 (6)	0.0308 (6)	0.0186 (6)	0.0005 (5)	0.0040 (5)	0.0010 (5)
S1	0.0216 (2)	0.0264 (2)	0.0123 (2)	0.00179 (13)	0.00437 (15)	−0.00110 (13)

### 2-Amino-5-(4-chlorophenyl)-1,3-thiazole-4-carbaldehyde (II) Geometric parameters (Å, º)

**Table:**

C2—N3	1.307 (2)	C8—C9	1.385 (3)
C2—N12	1.346 (2)	C8—H8	0.9500
C2—S1	1.7620 (16)	C9—C10	1.386 (3)
C4—C5	1.372 (2)	C9—Cl9	1.7410 (16)
C4—N3	1.386 (2)	C10—C11	1.387 (2)
C4—C13	1.448 (2)	C10—H10	0.9500
C5—C6	1.477 (2)	C11—H11	0.9500
C5—S1	1.7349 (16)	C13—O14	1.227 (2)
C6—C7	1.394 (2)	C13—H13	0.9500
C6—C11	1.400 (2)	N12—H12A	0.84 (3)
C7—C8	1.392 (2)	N12—H12B	0.81 (3)
C7—H7	0.9500		
			
N3—C2—N12	124.85 (15)	C8—C9—C10	121.58 (15)
N3—C2—S1	114.41 (12)	C8—C9—Cl9	119.62 (13)
N12—C2—S1	120.71 (13)	C10—C9—Cl9	118.80 (14)
C5—C4—N3	116.88 (15)	C9—C10—C11	119.03 (16)
C5—C4—C13	123.91 (15)	C9—C10—H10	120.5
N3—C4—C13	119.15 (14)	C11—C10—H10	120.5
C4—C5—C6	129.71 (15)	C10—C11—C6	120.57 (16)
C4—C5—S1	108.86 (12)	C10—C11—H11	119.7
C6—C5—S1	121.23 (12)	C6—C11—H11	119.7
C7—C6—C11	119.25 (15)	O14—C13—C4	123.35 (15)
C7—C6—C5	121.58 (15)	O14—C13—H13	118.3
C11—C6—C5	119.15 (15)	C4—C13—H13	118.3
C8—C7—C6	120.52 (16)	C2—N3—C4	110.36 (14)
C8—C7—H7	119.7	C2—N12—H12A	114.3 (18)
C6—C7—H7	119.7	C2—N12—H12B	116.8 (18)
C9—C8—C7	119.04 (16)	H12A—N12—H12B	119 (2)
C9—C8—H8	120.5	C5—S1—C2	89.48 (8)
C7—C8—H8	120.5		
			
N3—C4—C5—C6	173.71 (16)	Cl9—C9—C10—C11	179.63 (14)
C13—C4—C5—C6	−9.2 (3)	C9—C10—C11—C6	0.2 (3)
N3—C4—C5—S1	−1.08 (18)	C7—C6—C11—C10	−0.3 (3)
C13—C4—C5—S1	176.03 (13)	C5—C6—C11—C10	−178.47 (16)
C4—C5—C6—C7	128.1 (2)	C5—C4—C13—O14	179.28 (16)
S1—C5—C6—C7	−57.7 (2)	N3—C4—C13—O14	−3.7 (3)
C4—C5—C6—C11	−53.7 (3)	N12—C2—N3—C4	178.02 (16)
S1—C5—C6—C11	120.52 (16)	S1—C2—N3—C4	0.09 (18)
C11—C6—C7—C8	0.5 (3)	C5—C4—N3—C2	0.7 (2)
C5—C6—C7—C8	178.71 (16)	C13—C4—N3—C2	−176.60 (15)
C6—C7—C8—C9	−0.7 (3)	C4—C5—S1—C2	0.89 (12)
C7—C8—C9—C10	0.6 (3)	C6—C5—S1—C2	−174.43 (14)
C7—C8—C9—Cl9	−179.35 (13)	N3—C2—S1—C5	−0.59 (13)
C8—C9—C10—C11	−0.3 (3)	N12—C2—S1—C5	−178.61 (15)

### 2-Amino-5-(4-chlorophenyl)-1,3-thiazole-4-carbaldehyde (II) Hydrogen-bond geometry (Å, º)

**Table:**

*D*—H···*A*	*D*—H	H···*A*	*D*···*A*	*D*—H···*A*
N12—H12*A*···N3^i^	0.84 (3)	2.19 (3)	3.015 (2)	167 (2)
N12—H12*A*···O14^i^	0.84 (3)	2.61 (3)	3.1222 (19)	121 (2)
N12—H12*B*···O14^ii^	0.81 (3)	2.18 (3)	2.940 (2)	159 (2)
C9—Cl9···*Cg*1^iii^	1.74 (1)	3.53 (1)	4.5317 (19)	114 (1)

Symmetry codes: (i) −*x*+1, −*y*+1, −*z*; (ii) *x*+1/2, −*y*+1, *z*; (iii) *x*, −*y*+3/2, *z*+1/2.
