# Supplementary material for: Consistent supra­molecular motifs and different local symmetries in the structures of 2-amino-5-(4-fluoro­phen­yl)-1,3-thia­zole-4-carbaldehyde and 2-amino-5-(4-chloro­phen­yl)-1,3-thia­zole-4-carbaldehyde
Source: Acta Crystallogr E Crystallogr Commun. 2026 Jan 1;82(Pt 1):14–8. doi: 10.1107/S2056989025010667 (PMC12810316; doi:10.1107/S2056989025010667)
Supplement: Supplementary file 6 [file e-82-00014-sup6.pdf]

Supplementary Material

Crystal structures and Hirshfeld surface analyses of 2-amino-5-(4-fluorophenyl)-1,3-thiazole-4-carbaldehyde and 2-amino-5-(4-chlorophenyl)-1,3-thiazole-4-carbaldehyde

Firudin I. Guseinov,[a] Ksenia A. Afanaseva,[a] Sergey M. Gaidar,[b] Anna M. Pikina,[b] Mehmet Akkurt,[c] Fargana S. Aliyeva,[d] Khudayar I. Hasanov[e] and Alebel N. Belay[f]*

[a]Kosygin State University of Russia, 117997 Moscow, Russian Federation, and, N.D. Zelinsky Institute of Organic Chemistry, Russian Academy of Sciences, 119991 Moscow, Russian Federation, [b]Russian State Agrarian University–Moscow Timiryazev Agricultural Academy, 127550 Moscow, Russian Federation, [c]Department of Physics, Faculty of Sciences, Erciyes University, 38039 Kayseri, Turkey, [d]Excellence Center, Baku State University, Z. Xalilov Str. 23, Az 1148 Baku, Azerbaijan, [e]Azerbaijan Medical University, Scientific Research Centre (SRC), A. Kasumzade St. 14. AZ 1022, Baku, Azerbaijan, and [f]Department of Chemistry, Bahir Dar University, P.O.Box 79, Bahir Dar, Ethiopia

Correspondence e-mail: alebel.nibret@bdu.edu.et

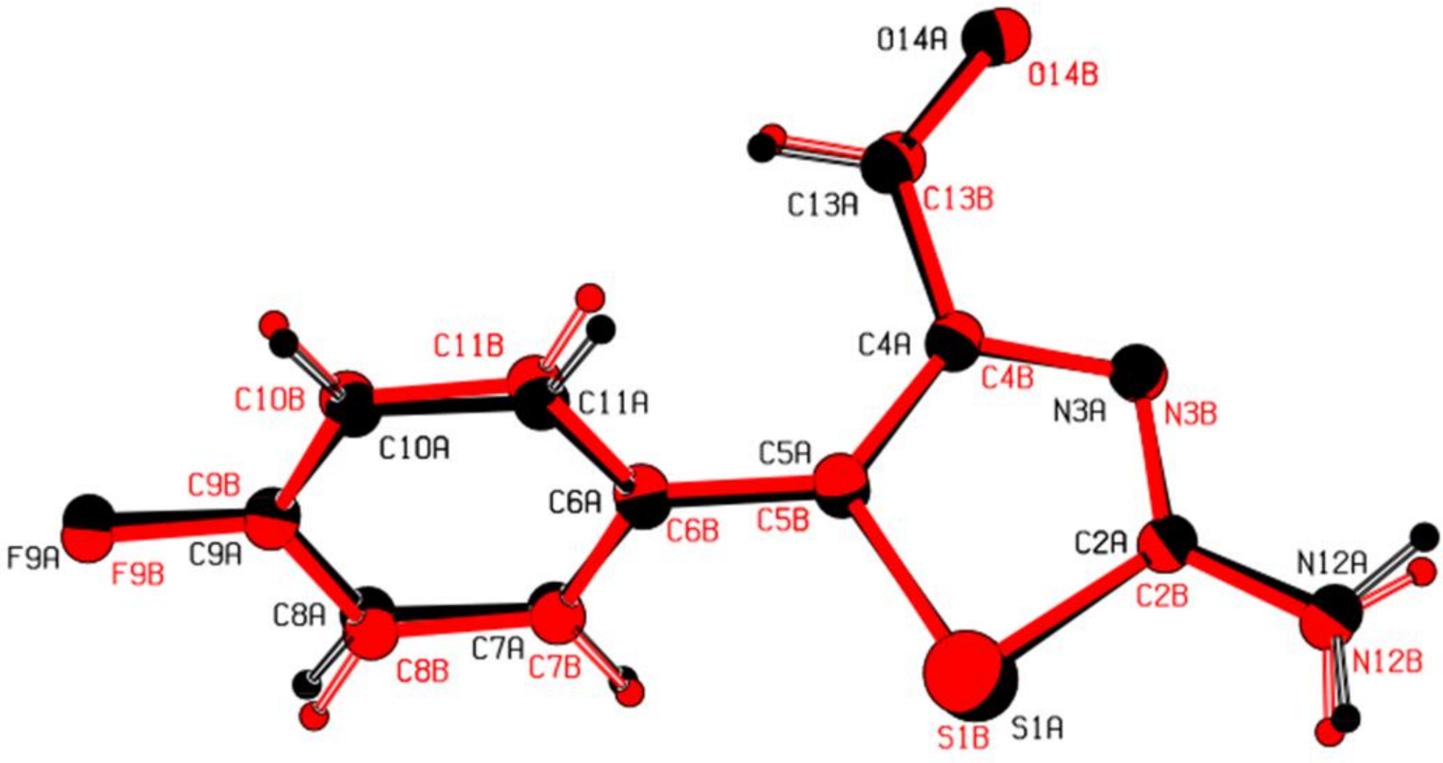

Figure S1 A least-squares overlay of the two independent molecules **A** and **B** [inverted molecule **B**(red) on molecule **A**(black) of (**I**)].

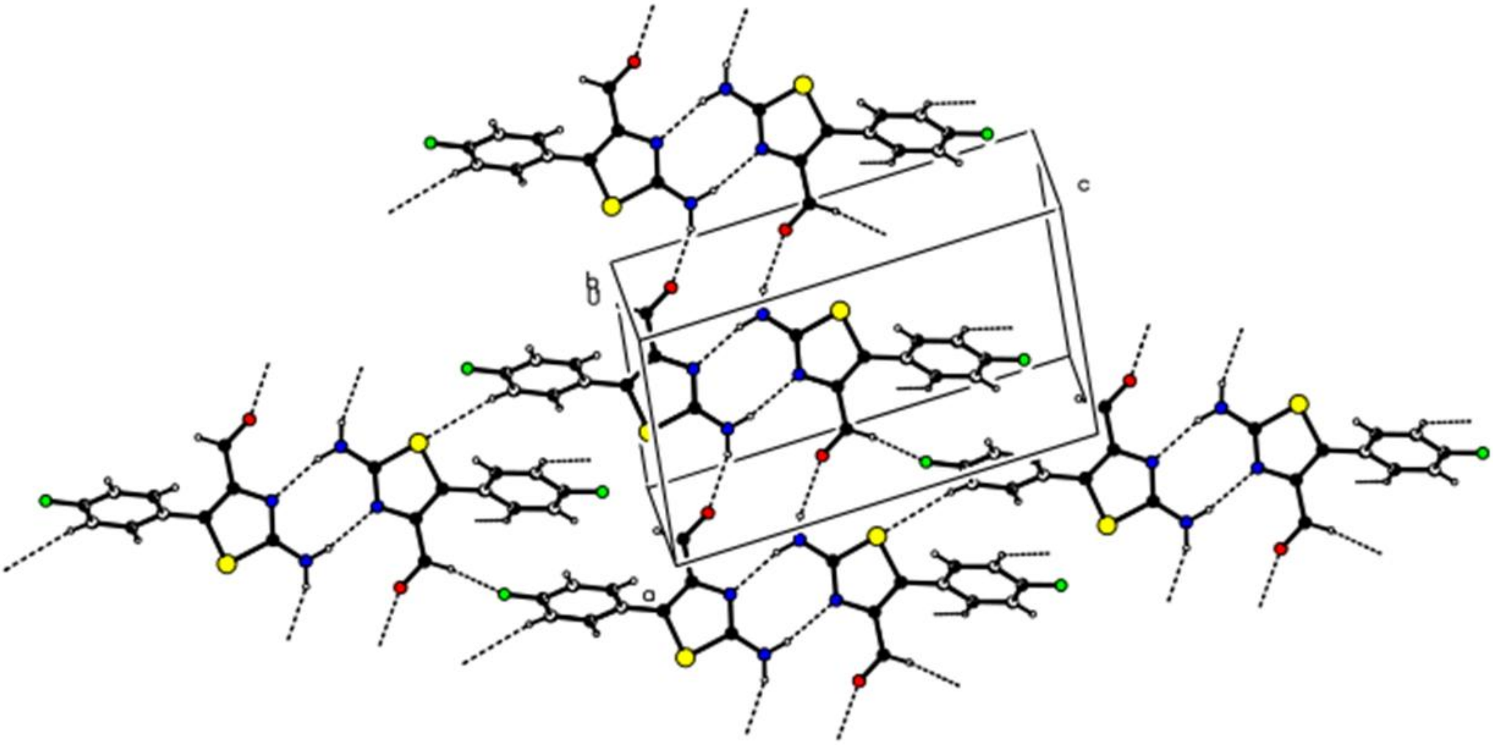

Figure S2 The molecular packing of (**I**), viewed down the *b*-axis, showing N—H···N, N—H···O, C—H···S, C—H···N and C—H···F hydrogen bonds.

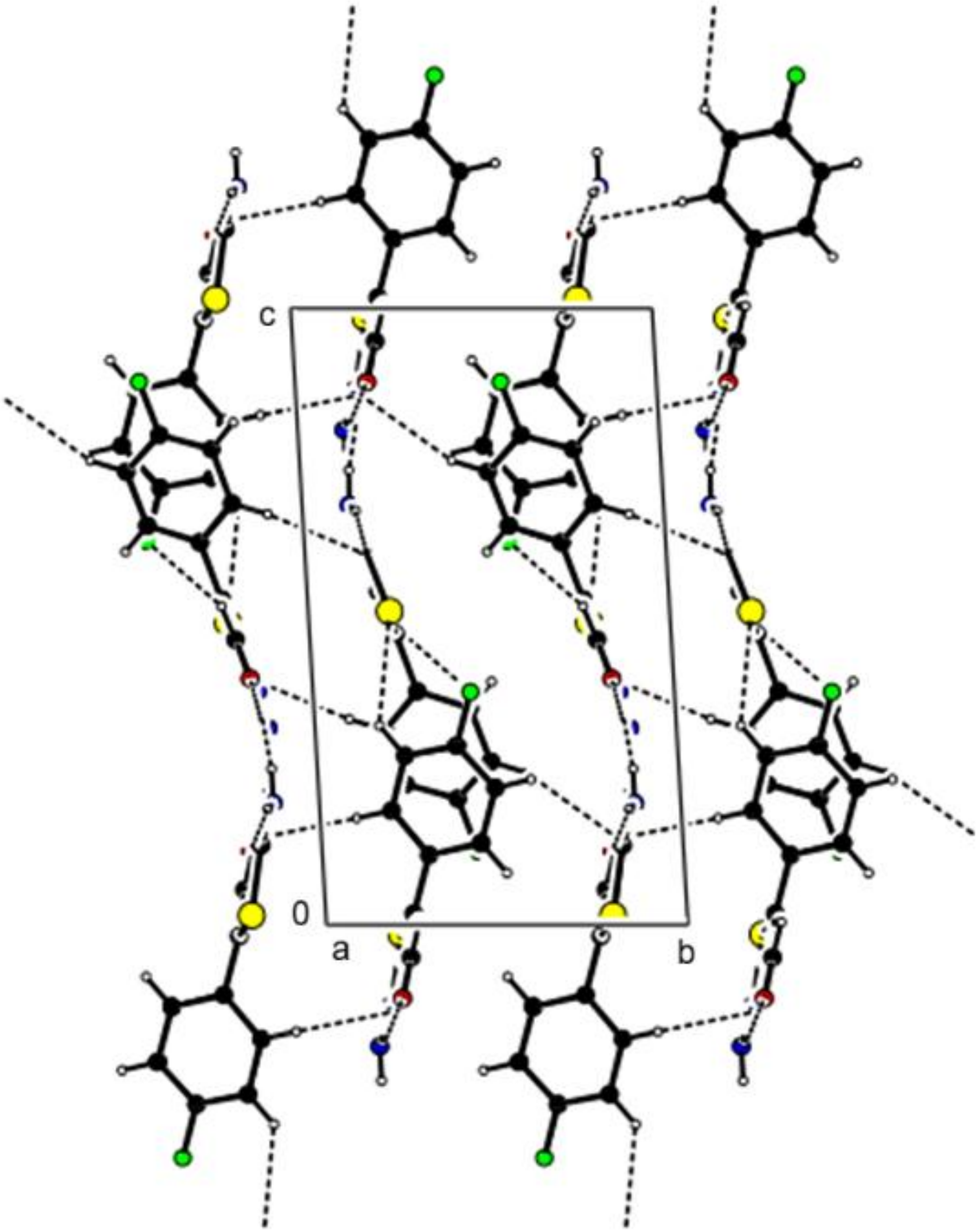

Figure S3 The molecular packing of (**I**), viewed down the *a*-axis, showing N—H···N, N—H···O, C—H···S, C—H···N and C—H···F hydrogen bonds.

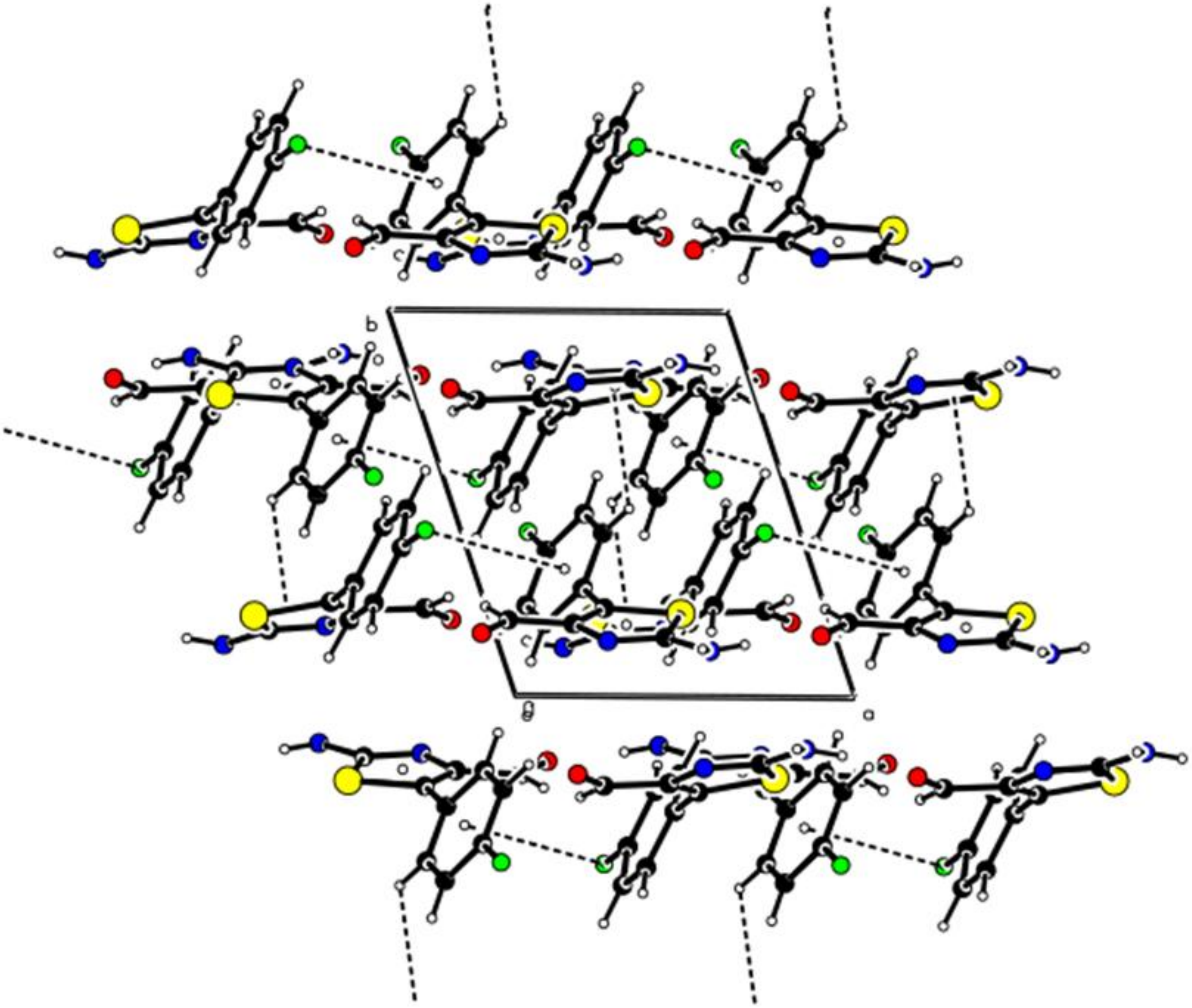

Figure S4 View down the *c*-axis of the C—H···π and C—F···π interactions (dashed lines) in (**I**).

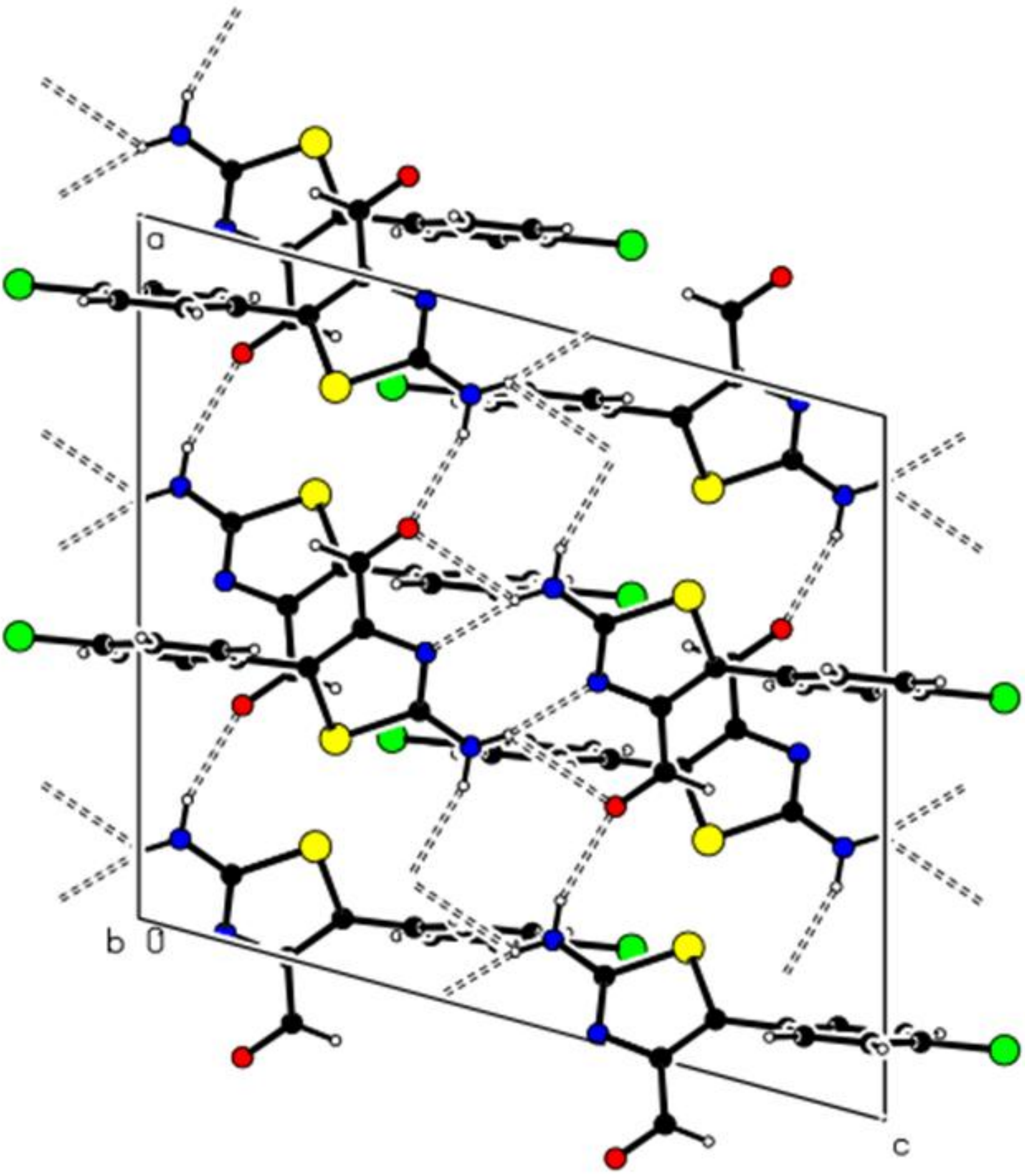

Figure S5 The molecular packing of (**II**), viewed down the *b*-axis, showing N—H···N and N—H···O hydrogen bonds.

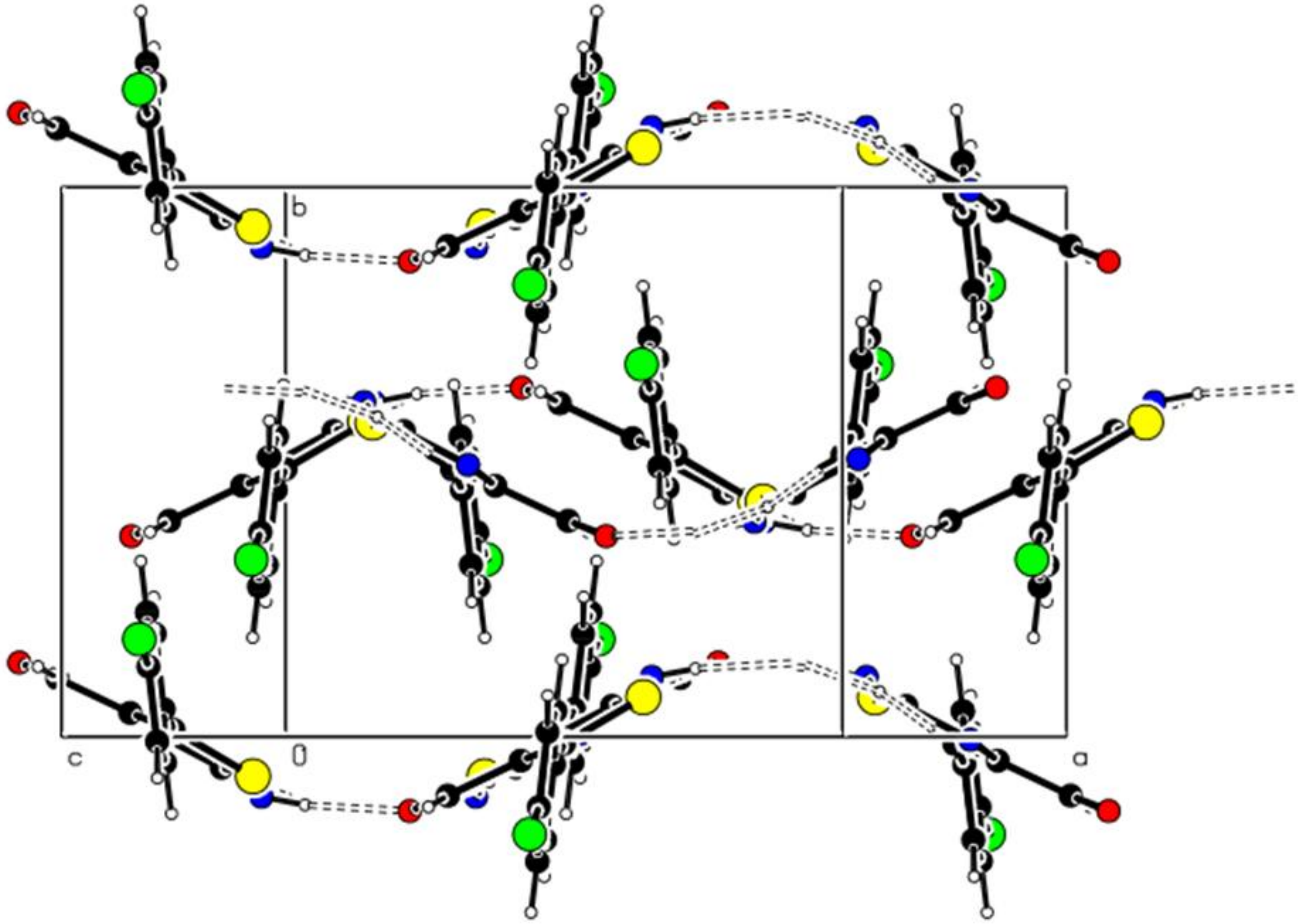

Figure S6 The molecular packing of (**II**), viewed down the *c*-axis, showing N—H···N and N—H···O hydrogen bonds.

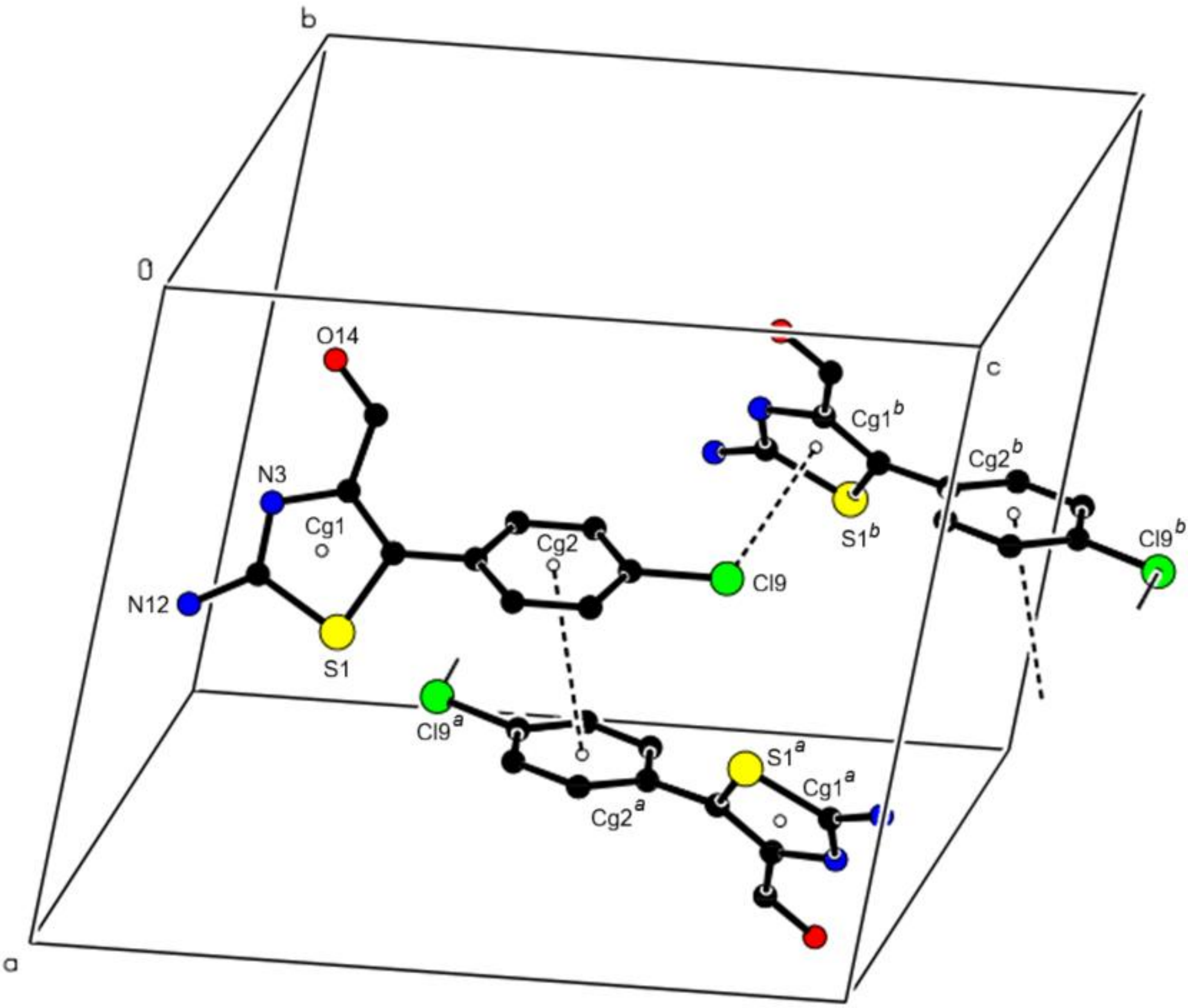

Figure S7 View in the unitcell of the π-π and C—Cl···π interactions (dashed lines) in (**II**). Symmetry codes: (a) 3/2-x, y, 1-z; (b) x, 3/2-y, 1/2+z.
